# Single cell analyses reveal the PD-1 blockade response-related immune features in hepatocellular carcinoma

**DOI:** 10.7150/thno.95971

**Published:** 2024-06-01

**Authors:** Yao Li, Fengwei Li, Lei Xu, Xiaodong Shi, Hui Xue, Jianwei Liu, Shilei Bai, Yeye Wu, Zhao Yang, Feng Xue, Yong Xia, Hui Dong, Feng Shen, Kui Wang

**Affiliations:** 1 Department of Hepatic Surgery II, Eastern Hepatobiliary Surgery Hospital, Naval Medical University, Shanghai, China.; 2 Department of Gastroenterology Nanjing Drum Tower Hospital, The Affiliated Hospital of Nanjing University Medical School, Nanjing, Jiangsu Province, China.; 3 Department of Hepatic Surgery IV, Eastern Hepatobiliary Surgery Hospital, Naval Medical University, Shanghai, China.; 4 Department of Pathology, Eastern Hepatobiliary Surgery Hospital, Naval Medical University, Shanghai, China.

**Keywords:** hepatocellular carcinoma, single cell, immune microenvironment, immunotherapy, PD-1

## Abstract

**Background:** Immunotherapy has demonstrated its potential to improve the prognosis of patients with hepatocellular carcinoma (HCC); however, patients' responses to immunotherapy vary a lot. A comparative analysis of the tumor microenvironment (TME) in responders and non-responders is expected to unveil the mechanisms responsible for the immunotherapy resistance and provide potential treatment targets.

**Methods:** We performed sequencing analyses using 10x Genomics technology on six HCC patients who responded to anti-PD-1 therapy and one HCC patient who did not respond. Additionally, we obtained single cell data from untreated, responsive, and nonresponsive HCC patients from public databases, and used part of the datasets as a validation cohort. These data were integrated using algorithms such as Harmony. An independent validation cohort was established. Furthermore, we performed spatial transcriptomic sequencing on the tumor adjacent tissues of three HCC responsive patients using 10x Genomics spatial transcriptomic technology. Additionally, we analyzed data about three HCC patients obtained from public databases. Finally, we validated our conclusions using immunofluorescence, flow cytometry, and *in vivo* experiments.

**Results:** Our findings confirmed the presence of “immune barrier” partially accounting for the limited efficacy of immunotherapy. Our analysis revealed a significant increase in TREM2^+^ Macrophages among non-responsive patients expressing multiple immunosuppressive signals. anti-Csf1r monoclonal antibodies effectively eliminated these macrophages and augmented the therapeutic effects of anti-PD-1 therapy. TCR^+^ Macrophages possessed direct tumor-killing capabilities. IL1B^+^ cDC2 was the primary functional subtype of cDC2 cells. Absence of THEMIS^hi^ CD8^+^ T subtypes might diminish immunotherapeutic effects. Furthermore, CD8^+^ T cells entered a state of stress after anti-PD-1 treatment, which might be associated with CD8^+^ T cell exhaustion and senescence.

**Conclusions:** The profiles of immune TMEs showed differences in HCC patients responsive, non-responsive and untreated. These differences might explain the discounted efficacy of immunotherapy in some HCC patients. The cells and molecules, which we found to carry unique capabilities, may be targeted to enhance immunotherapeutic outcomes in patients with HCC.

## Introduction

Hepatocellular carcinoma (HCC) is the third leading cause of cancer-related mortality 1, with a five-year survival rate nearing 18% 2. Immunotherapy has transformed the landscape of HCC treatments. In a double-blind, phase III trial, pembrolizumab, as a second-line treatment, significantly increases the median overall survival in patients with advanced HCC, compared to the placebo group; however, the objective response rate (ORR) is only 12.7% in the pembrolizumab group 3. The outcomes achieved in the IMBrave150 study may herald an era of combination immunotherapy; nevertheless, the ORR remains at 30% in patients with unresectable advanced HCC 4. Besides, a considerable proportion of HCC patients still exhibit suboptimal responses to immunotherapy, necessitating a comprehensive analysis of underlying cellular and molecular mechanisms.

The suboptimal efficacy of immunotherapy can be attributed to several factors, including inadequate T cell infiltration and activation 5, low mutation burden 6, and presence of immunosuppressive cells in the microenvironment (TME) 7. The infiltration and activation of CD8^+^ T cells within the TME are crucial determinants for the efficacy of immunotherapy in breast and oral cancers 8, 9. The activation of intratumoral CD8^+^ T cells is influenced by various factors, particularly the level of conventional type 1 dendritic cells (cDC1s) that activate CD8^+^ T cells via antigen cross-presentation 10. Studies have also highlighted the significant role of CCR7 negative cDC1s in promoting CD8^+^ T cell proliferation and activation 11. In a recent research, one end of a monoclonal antibody is bound to the CLEC9A surface marker on cDC1s, while its other to PD-1 blocking antibodies; this approach draws cDC1s closer to activate CD8^+^ T cells, thereby stimulating a stronger anti-tumor activity 12. Furthermore, the cDC2 subpopulation is a heterogeneous group with an unclear role in tumor development. Moreover, mregDCs exhibiting high LAMP3 expression and plasmacytoid dendritic cells (pDCs) in tumors have been recognized. In tumors, an immunosuppressive TME can be established by such cells as tumor-associated macrophages (TAMs), cancer-associated fibroblasts (CAFs), neutrophils, and regulatory T cells (Tregs) 10, 13, 14. Researchers have discovered that CD8^+^T cells and CD163^-^ Arg1^hi^ macrophages are spatially close in non-responders to PD-1 therapy, suggesting their impairment on PD-1 efficacy 15. In addition, spatial barriers within tumors significantly affect the effectiveness of immunotherapy. For example, SPP1^+^ Macrophages and CAFs collaborate to form a peripheral immune barrier that hinders the infiltration of CD8^+^ T cells. Interventions can be designed to facilitate the infiltration of T cells through this barrier, thus enhancing antitumor responses 16. Previous factors have also discovered an array of factors contributing to suboptimal immunotherapeutic responses in HCC 17, 18. However, the immune TMEs among responders, non-responders, and untreated patients remain to be further profiled.

In this study, we employed single cell sequencing technology and integrated data from public databases to comprehensively characterize myeloid and T cells associated with anti-PD-1 therapy in HCC, thereby offering new insights into the mechanisms underlying inadequate treatment responses.

## Materials and Methods

### Human subjects

This study enrolled 16 patients diagnosed with HCC at the Third Affiliated Hospital of Naval Medical University, and had obtained relevant ethical approval (registration number: EHBHKY2020-K-022). Before surgery, all patients were informed about the potential use of their pathological specimens for medical research, and all patients signed on informed consent forms. Before surgical intervention, three cycles of pembrolizumab were administered to all patients, and two associate chief physicians evaluated the treatment response based on the mRECIST criteria. Single-cell sequencing was conducted on the tumor, adjacent, and transition zone tissues from seven patients, and spatial transcriptomic analysis exclusively on transition zone tissues from three patients. Patients' information is detailed in the supplementary Table S1

### Preparation of single cell and spatial transcriptomics samples

Single-cell transcriptome samples were prepared using Chromium Single Cell 5' Reagent Kits V2 from 10x Genomics, following the manufacturer's instructions. The libraries were then sequenced. Cells from each patient were washed once with PBS containing bovine serum albumin (BSA) and resuspended in PBS containing 0.04% BSA at a final concentration of 500 to 1200 cells/mL. Approximately, 6000 to 10000 cells were captured using a cell counter to form nanogramme-scale GEMs. Reverse transcription was performed using a C1000 Touch Thermal Cycler (Bio-Rad) with the following program: incubation at 53 °C for 45 min followed by denaturation at 85 °C for 5 min and cooling to 4 °C. After completion of reverse transcription and cell barcoding steps, emulsion breakage was performed, and cDNA was purified using a Cleanup Mix comprising DynaBeads and SPRIselect Reagents (Thermo Fisher Scientific). Subsequent PCR amplification was conducted, in which amplified cDNA underwent fragmentation, end-repairing, and size-selection before PCR amplification using sample indexing primers. The PCR products generated during enrichment underwent further fragmentation, end-repair, and size selection, followed by another round of PCR amplification using sample indexing primers for a second time. Finally, libraries were prepared according to the manufacturer's instructions, and subjected to quality assessment and purification before sequencing.

### Single cell data quality control

To ensure the reliability of the data, we initially employed the DoubletFinder software to eliminate doublet cells. Subsequently, we utilized the PercentageFeatureSet function to quantify mitochondrial genes in each cell, only retaining cells with a percentage of mitochondrial genes (percent. mt) below 10%.

### Sc-RNA dimension reduction, clustering, and subtype identification

**Figure 1:**
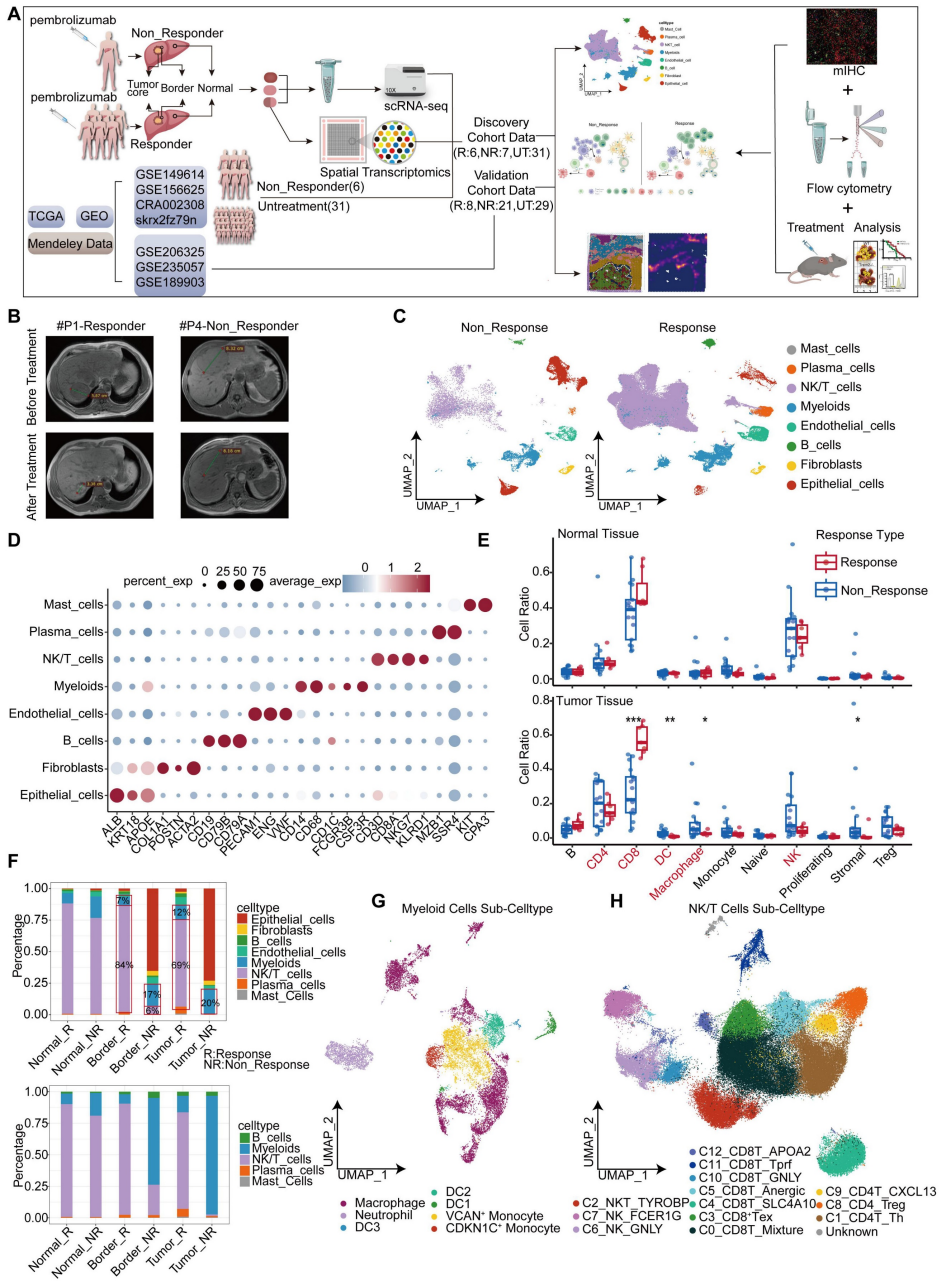
** Single cell atlas of HCC samples responsive and non-responsive to PD-1 blockade.** (A) Workflow of this study. (B) MRI images of patients with and without response to treatment. (C) Cell type maps for different response conditions. (D) Cell subgroups and their corresponding gene markers. (E) Box plots showing the proportions and statistics of various cell types in response and non-response patients in the GSE206325 cohort (Wilcoxon test. *P < 0.05, **P < 0.01, ***P < 0.001, ****P < 0.0001). (F) The proportion of cell types across different response types in tumor tissue with our cohort. (G) UMAP plot illustrating myeloid cell subpopulations. (H) UMAP plot showing NK/T cell subpopulations.

After data normalization, the FindVariableFeatures function was used to identify the top 2000 genes exhibiting the highest variability for subsequent principal component analysis (PCA). The RunHarmony function was employed to correct batch effects arising from multiple datasets. by. vars parameter set to patient and dataset. Dimensionality was then reduced to 1-30 using the RunUMAP function, specifying 'Harmony' as the reduction method. Cells were clustered using the FindClusters function with a resolution of 0.5. Cell subpopulations were identified based on markers specific to various cell types (Figure 1D).

### Trajectory analysis

Trajectory analysis of CD8^+^ T cells was performed using Monocle2 software (version 2.30.0). Differential gene expression (DGE) analysis was conducted using the Differential Gene Test function to identify significant genes (p-values < 0.01), which were then subjected to unsupervised cell ordering. Trajectory construction was completed following dimensionality reduction and default parameters for cell ordering. For other CD8^+^ and CD4^+^ T cells, trajectory analysis was performed using Monocle3 (version 1.3.4). Two-dimensional mapping coordinates were obtained by replacing int. embedded with UMAP coordinates after data dimensionality reduction.

### Cell-cell interaction analysis using Cellchat and CellphoneDB

Cell-cell communication was analyzed using the Cellchat and CellphoneDB, with p-values below 0.05 to determine receptors and signaling pathways. All other parameters were set at their default values. Receptors and signaling pathways were visualized using built-in functions in Cellchat and ggplot2.

### Spatial transcriptomics analysis

**Figure 2:**
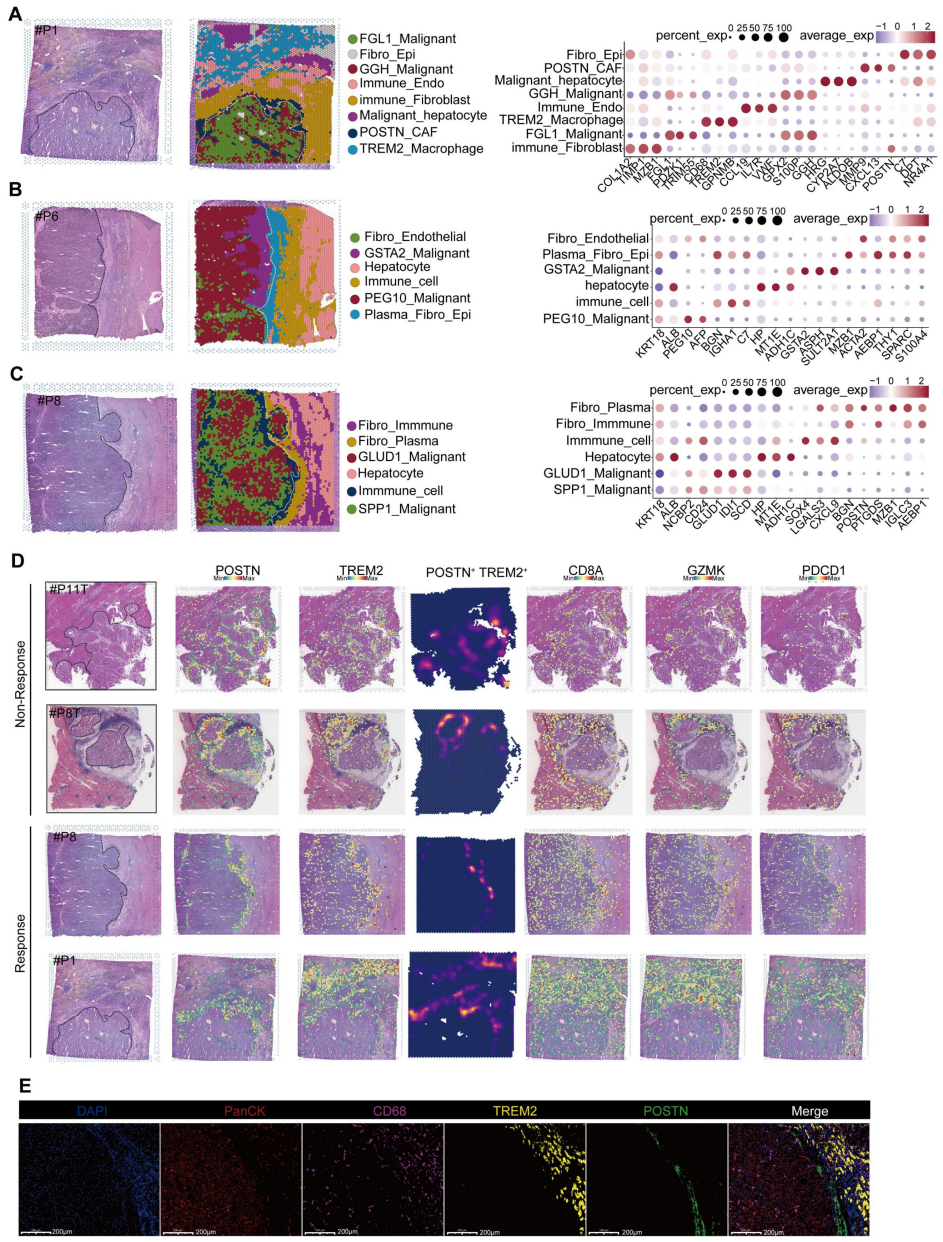
** Spatial transcriptomic features of responsive and non-responsive HCC adjacent tissues.** (A-C) Cell types and corresponding markers in patients P1, P6, and P8. (D) Distribution images of POSTN, TREM2, CD8A, GZMK, and PD-1 in the tumor margin of patients and density distribution maps of POSTN and TREM2 expression, indicating the presence of immune barriers in both responsive and non-responsive patients. (E) TREM2 and CD68 represent TREM2^+^ Macrophages, and POSTN represents POSTN^+^ CAFs). Multicolor immunofluorescence staining of the tumor margin in patient P8 further demonstrates the existence of immune barriers composed of TREM2^+^ Macrophages and POSTN^+^ CAFs in responsive patients.

All spatial transcriptomic data were processed using the Load10X_Spatial function, followed by data normalization using the SCTransform function. Spatial region clustering was conducted using the FindClusters function. Feature gene analysis for each cluster was performed using the FindAllMarkers function. To plot the density of the co-expressed gene regions (Figure 2D), spatial coordinates from spatial transcriptomics data were replaced with two-dimensional coordinates obtained from UMAP dimensionality reduction. Plots were generated using the Plot_density function.

### Survival analysis

Survival curves were plotted for patients in TCGA-LIHC and other cohorts using the Survival and Survminer R packages. In patient groups, the surv_cutpoint function was used to calculate the optimal cut-off values, which were then based on to subgroup the patients for survival curve plotting.

### Bulk RNA-seq analysis

Bulk RNA-seq data used in this study were sourced from The Cancer Genome Atlas Liver Hepatocellular Carcinoma (TCGA-LIHC), International Cancer Genome Consortium-Liver Cancer (ICGC-LINC-JP), and GSE14520 (Gene Expression Omnibus). The data were subjected to TPM (Transcripts Per Million) normalization before downstream analyses, including survival analysis, gene set enrichment analysis, and other related analyses.

### Gene bubble plot

Feature genes for subpopulations were calculated using the FindMarkers function and plots were generated using the ggplot2 R package. The Diff parameter for results from FindMarkers was calculated as follows: Diff = pct.1 - pct.2.

### Cell subpopulation similarity analysis

After standard procedures were applied to the target cell subpopulation of the Seurat object, the FindTransferAnchors function was used to search for anchors, with cell subpopulations in the discovery queue as references. Similarity between the anchors was predicted using the TransferData function. Finally, the average similarity score for a single-cell subpopulation was calculated and used as the similarity score. More detailed methods could be consulted at PMID: 35325594, section “method”.

### Cell type propensity analysis

We referred to previously published literature (PMID: 37248301) for cell types and their corresponding characteristic genes, and utilized the Aucell algorithm to profile individual cells based on the scores of these gene sets. Subsequently, we calculated the mean score of all the cells in one subgroup. The results were visualized using radar plots. Detailed information regarding the relevant gene sets are found in the Supplementary Files, specifically in Table S7 and Table S8.

### Flow cytometry data analysis and visualization

Fresh surgical specimens were minced into rice-sized tissue pieces using scissors, followed by tissue dissociation according to instructions provided with the Human Tumor Dissociation Kit (Miltenyi Biotec #130-095-929). Subsequently, the dissociated single-cell suspension was filtered through a 40 μm mesh. Resultant single-cell suspension was incubated with antibodies at 4 °C using Live-Death (BD, Cat#564406), CD68 (INVITROGEN, Cat#2473661), TREM2 (RD, Cat#FAB17291A), and CD45 (BD, Cat#557659) for 30 min, centrifuged again at 500 g and resuspended in staining buffer before being loaded onto a flow cytometer. The acquired data were analyzed using FlowJo software.

### Multiplex immunofluorescence

Multiplex immunofluorescence staining was performed using a PANO 7-plex IHC Kit (Panovue, Cat#0004100100). Primary antibodies were sequentially applied, followed by incubation with horseradish peroxidase-conjugated secondary antibodies. Tyramide signal amplification (TSA) allowed the acquisition of multiple immunofluorescent markers. After each TSA step, the slides were heated with a microwave. Following labelling with all human antigens, cell nuclei were stained with 4,6-diamidino-2-phenylindole (DAPI). To obtain multispectral images, stained slides were scanned using the Mantra System (PerkinElmer). Fluorescence spectra were captured at 20 nm wavelength intervals from 420 to 680 nm under identical exposure periods. The scanned images were combined to construct a single-stacked image. The extracted images were further utilized to build the spectral library required for multispectral unmixing, using the InForm Software (SlideViewer). Related antibodies used in this study included TREM2 (RD, Cat#MAB17291), CD68 (abcam, Cat#ab289671), PanCK (abcam, Cat#ab234297), POSTN (proteintech, Cat#66491-1-lg), CD3 (abcam, Cat#ab16669), CLEC10A (abcam, Cat#ab315086), HSPA1B (proteintech, Cat#10995-1-AP), DPYD (proteintech, Cat#27662-1-AP), IL1B (proteintech, Cat#16806-1-AP) and THEMIS (proteintech, Cat#27415-1-AP).

### Animal experiments

All animals used in this study were housed in an SPF environment.

All the HCC mouse models used in this study were created using the Sleeping Beauty transposon system. The mice aged 5-6 weeks were injected with 25 μg Nras, 25 μg c-Myc, and 2 μg Sleeping Beauty plasmid via high-pressure hydrodynamic injection from the tail vein. Liver cancer was allowed to spontaneously develop between weeks 10-11, during which drugs were administered. After treatment, the mice were sacrificed through dislocation of the cervical vertebrae, and the following parameters were compared between the experimental and control mice: the number of tumors in each mouse, the weight of the liver, and the liver-to-body weight ratio. The plasmid used in this study was provided by Professor Wang Lei's research team at the Department of Gastroenterology, Nanjing University, Drum Tower Hospital, China. Wild-type mice were purchased from Suzhou Saiye Biological Experimental Animal Co., Ltd., and C57BL/6Smoc-*Trem2^em1Smoc^* (catalog number: NM-KO-190402 (https://www.modelorg.com/portal/article/index/id/3555/post_type/3.html)) was purchased from Shanghai Southern Model Organisms Co., Ltd.

The mouse was treated from week 7 after the model was established. anti-Csf1r monoclonal antibodies were administered at a dose of 400 μg/per mouse per injection, anti-PD-1 monoclonal antibodies at a dose of 200 μg/per mouse per injection, and isotype antibodies at a dose of 250 μg/per mouse per injection. The combined treatment group received anti-PD-1 monoclonal antibodies (200 μg/per mouse) plus anti-Csf1r monoclonal antibodies (400 μg/per mouse). The injection volume was set at 0.5 mL per mouse per injection. Antibodies were injected every 3 days for a total of 7 times. AAV-Themis and scrambled short hairpin RNA (shRNA) were purchased from Jimin Biotech Co., Ltd. (Shanghai) Co., China). AAV was diluted in physiological saline to 1.5×10^12^ copies/mL, and for each mouse, every 0.1 mL was injected via the tail vein daily for 12 consecutive days. anti-PD-1 monoclonal antibodies (InVivoMab, Cat#BE0273), anti-Csf1r monoclonal antibodies (InVivoMab, Cat#BE0213), and mouse IgG antibodies (YEASEN, Cat#36111ES60) were used.

## Results

### Single cell atlas of HCC samples responsive and non-responsive to PD-1 blockade

In this study, we constructed a high-resolution map at the single cell level to depict the heterogeneity in the response and resistance of HCC toward anti-PD-1 therapy. Our conclusions were validated in independent cohorts and mouse experiments (Figure 1A). Seven HCC patients were included. Their tumor, peritumoral (border), and adjacent non-tumor (normal) liver tissues were collected (Table S1). Among these patients, one exhibited resistance to PD-1 blockade, with inconsistent MRI results before and after treatment (Figure 1B). We employed the 10x Genomics single cell technology for comprehensive scRNA-seq analysis of 21 samples, and performed spatial transcriptomic analysis of three samples selected from these individuals. Owing to the limited number of responders in our cohort, we incorporated data sourced from publicly accessible databases, including scRNA-seq data from six patients and spatial transcriptomics data from three patients. Three untreated HCC cohorts were included as controls (Table S1). We delineated the single-cell landscape of the seven patients, and classified them into eight distinct cell types (Figure 1C, Figure S1A). The unique molecular features of each cell type were characterized (Figure 1D). Notably, patient 4 (P4), who exhibited resistance to treatment, demonstrated a proportion of epithelial cells exceeding 50% (Figure S1B-C). These epithelial cells primarily originated from the tumor and border regions (Figure S1D-E).

Owing to the substantial disparity in patient numbers, we performed a statistical analysis of the proportions of different cell types using an independent dataset (GSE206325) (Figure S1F). Our findings revealed higher proportions of CD4^+^ T cells, dendritic cells (DCs), macrophages, stromal cells, and regulatory cells (Tregs) in non-response tumor tissues (Figure 1E). However, only DCs, stromal cells, and macrophages showed significant differences between responsive and non-responsive HCC tissues. Notably, the proportion of CD8^+^ T cells was significantly elevated in responsive HCC tissues, but did not exhibit similar patterns in normal tissue (Figure 1E). Our analogous analysis within our study cohort identified significant variations in the proportions of myeloid and NK/T cell types (Figure 1F). A remarkably low proportion of NK/T cells was observed in non-responsive patients (P4), both tumor and border tissues, compared to responsive patients; however, this proportion was similar to that observed in normal tissue (Figure S1E). Consequently, we analyzed myeloid and NK/T cells. As to myeloid cells, we characterized macrophages along with three distinct dendritic cell subtypes, two monocyte subtypes, and a unique neutrophil subtype (Figure 1G), using specific markers (Figure S1G). The analysis of NK/T cells revealed two NK cell subtypes, three CD4^+^ T cell subtypes, seven CD8^+^ T cell subtypes, and one NKT cell subtype (Figure 1H, Figure S1H).

### Spatial transcriptomic features of responsive and non-responsive HCC adjacent tissues

We performed spatial transcriptomic sequencing of HCC adjacent tissues obtained from three patients (Table S1). Additionally, we included the spatial transcriptomic data from three patients provided by Liu et al. 16 for secondary analysis. Among the six patients analyzed, four exhibited positive responses to anti-PD-1 therapy (P1, P6, P8, and P9T), whereas two were non-responders (P8T and P11T) (Figure 2A-C; Figure S2A-C). Notably, significant heterogeneity was observed in the distribution of tumor cell types across different patients; however, normal hepatocytes were more concentrated (Figure S2D). These findings were further supported by a similarity analysis (Figure S2E). Interestingly, despite the interpatient heterogeneity in tumor cell distribution, two-layer structures were identified in tumor cells from patients P11T, P1, and P8. Further investigations are required to elucidate the mechanisms underlying these structures.

As proposed by Liu et al., “immune barrier”, composed of SPP1^+^ Macrophages and CAFs, impedes T cell infiltration, thereby influencing the efficacy of immunotherapy 16. In addition to spatial transcriptomics, other new methods like the spQSP have also shown that CD8^+^ T cells and macrophages are more closely distributed in HCC patients resistant to a combination of targeted treatment and immunotherapy 19. TREM2 was predominantly expressed by macrophages and POSTN by CAFs, thus creating an immunosuppressive TME 20-23. Using TREM2 and POSTN as markers (Figure 2D), we confirmed the existence of immune barriers in both responders and non-responders. Immunofluorescence staining of border tissues from patient P8 provided evidence supporting our observations (Figure 2E). Notably, two responsive patients (P6 and P9T) did not exhibit features of immune barriers.

Further comparisons revealed significant difference in CD8^+^ T cell population between responsive and non-responsive patients. Responsive patients exhibited a significantly higher number of CD8^+^ T cells, with P8 showing a predominant distribution of CD8^+^ T cells within the tumor tissue, whereas P8T and P11T in the marginal regions. In contrast, CD8^+^ T cells in P1 were distributed within the immune cell reaction area, accompanied by the infiltration of some CD8^+^ T cells into the tumor interior. Overall, responsive patients demonstrated a higher abundance of CD8^+^ T cells, highlighting that the immunotherapeutic efficacy of anti-PD-1 therapy is influenced by various factors, including spatial structure and the status (cold or hot) of tumors 24.

### TREM2^+^ Macrophages represent a predominant immunosuppressive subset within the macrophage population

Single cell technology has uncovered the pivotal role of myeloid cell subpopulations in the innate immunity 25. Here, we analyzed the subpopulations of myeloid cells (Figure 1G). Neutrophils can be characterized by high expression of FCGR3B 26, one monocyte subpopulation by high expression of VCAN, EREG, and IL1B, and another by high expression of CDKN1C.

Macrophages are marked by CD68, MRC1, and CD163 (Figure S3A) 25. Moreover, investigations have revealed three DC types: DC1 cells expressing prominent CLEC9A, as the cDC1 subtype responsible for antigen presentation to CD8^+^ T lymphocytes; DC2 cells characterized by increased expression of CLEC10A and FCGR1A, as the cDC2 subtype primarily interacting with CD4^+^ T cells; and an additional subpopulation marked by high LAMP3 and CCR7 expression (Figure S3A) 25, capable of homing to lymph nodes that may be crucial for T lymphocyte activation within these nodes 27. The proportion of macrophages increased gradually from the normal tissue, to the tumor boundary, and then to the core region, whereas the proportions of neutrophils, along with DC1, DC2, and CDKNIC^+^ monocytes, gradually decreased (Figure S3B, S3C). These alterations suggest that a flourish of macrophages coupled with a lack of DCs in the tumor tissue might contribute to tumor progression.

**Figure 3:**
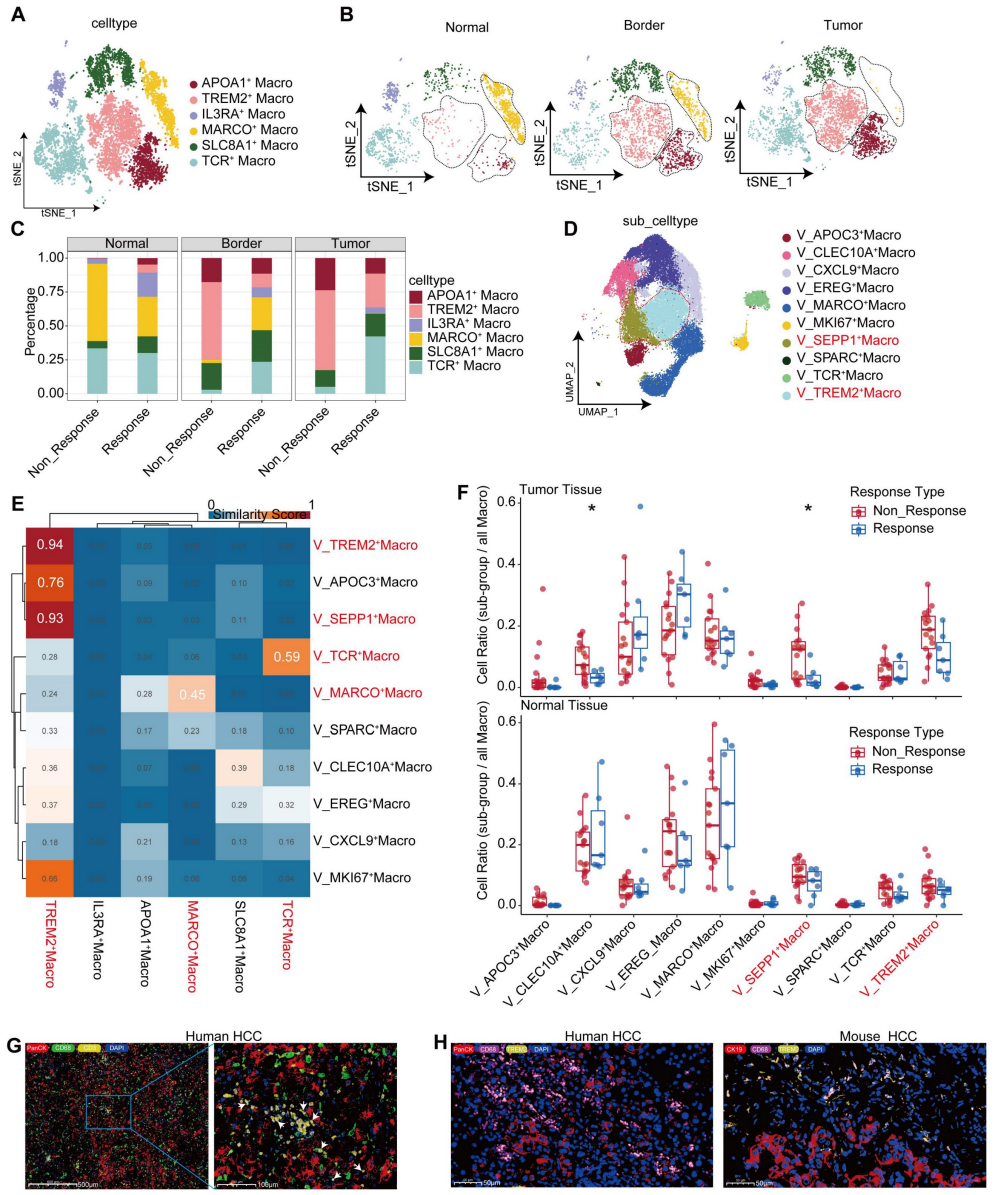
** TREM2^+^ Macrophages represent a predominant immunosuppressive subset within the macrophage population.** UMAP plot illustrating subpopulations of macrophages in our cohort. (B) UMAP plot showing subpopulations of macrophages in different tissue sites. (C) Bar graph depicting the distribution of macrophage subgroups across different tissue types. (D) UMAP plot illustrating subpopulations of macrophages in the GSE206325 cohort. (E) Similarities between macrophage subpopulations in our cohort and the GSE206325 cohort. (F) Boxplot showing the distribution of macrophage subpopulation proportions in treatment response type and tissue type (Wilcoxon test. *P < 0.05, **P < 0.01, ***P < 0.001, ****P < 0.0001). (G) Multicolor immunofluorescence showing TCR^+^ Macrophages in HCC-mouse module tumor tissue. (H) Multicolor immunofluorescence showing TREM2^+^ Macrophages in human HCC tumor tissue.

**Figure 4:**
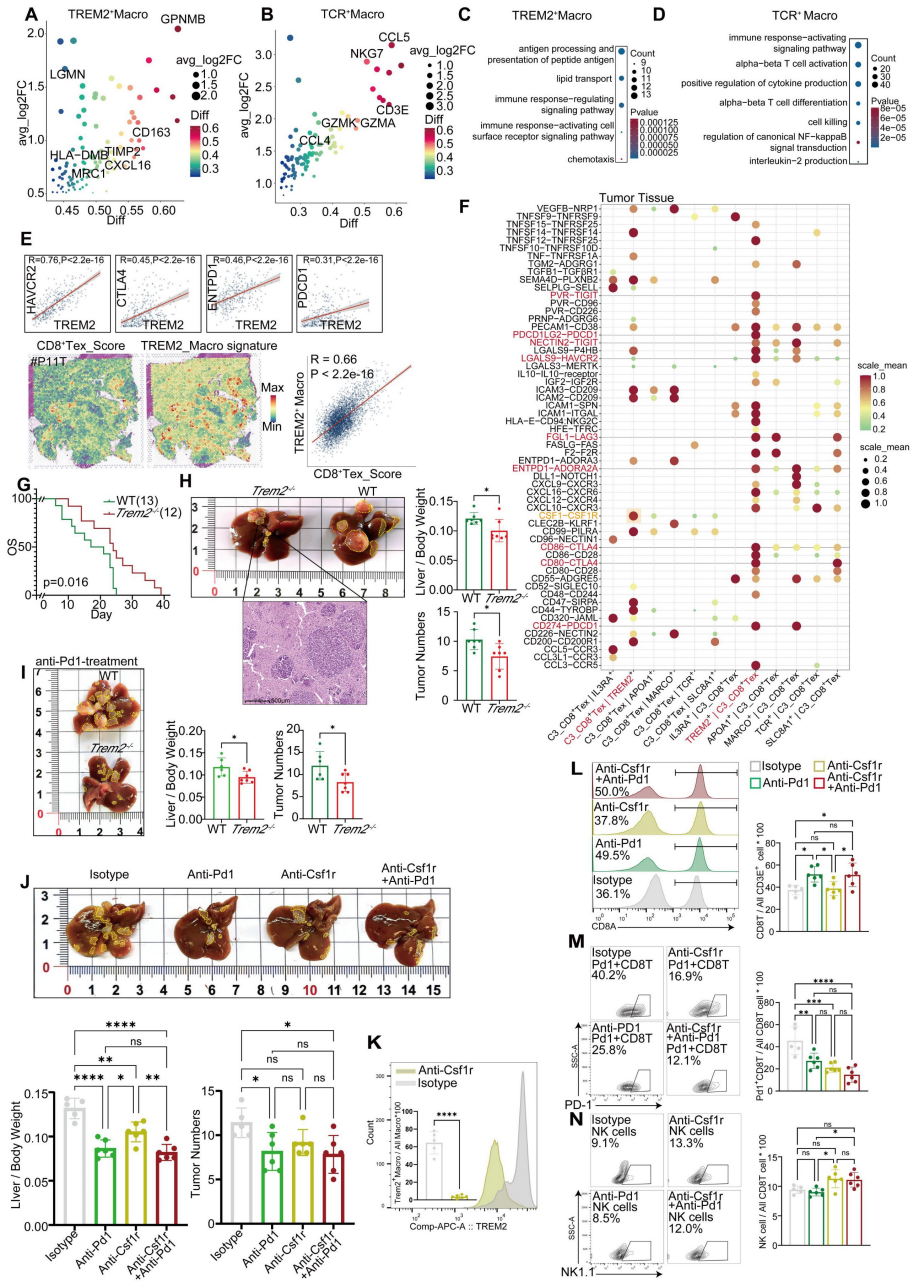
** TREM2^+^ macrophages represent a predominant immunosuppressive subset within the macrophage population.** Marker genes of TREM2^+^ Macrophages (top50). (B) Marker genes of TCR^+^ Macrophages (top50). (C) GO enrichment results for the signature gene of TREM2^+^ Macrophages. (D) GO enrichment results for the signature gene of TCR^+^ Macrophages. (E) Correlation of TREM2 expression with HAVCR2, CTLA4, PDCD1, ENTPD-1 in the TCGA-LIHC cohort (analyzed using Log2(TPM+1) values) and spatial correlation of TREM2 expression with CD8^+^ Tex signatures in P11T. (F) The dot-plot shows the results of CD8^+^ Tex and macrophage subgroups pair-receptor combinations calculated by CellphoneDB software in the tumor tissue of our cohort (after filtering with p-values < 0.05 condition). (G) Kaplan-Meier survival analysis *Trem2^-/-^* and WT HCC-mouse module. (H) tumor numbers and liver-body weight ratio in *Trem2^-/-^* and WT HCC-mouse module (T-test. *P < 0.05, **P < 0.01, ***P < 0.001, ****P < 0.0001). (I) tumor numbers and liver-body weight ratio in *Trem2^-/-^* and WT HCC-mouse module after treated with anti-PD-1 (T-test. *P < 0.05, **P < 0.01, ***P < 0.001, ****P < 0.0001). (J) tumor numbers and liver-body weight ratio in *Trem2^-/-^* and WT HCC-mouse module after treated under different conditions (T-test. *P < 0.05, **P < 0.01, ***P < 0.001, ****P < 0.0001). (K) The difference of TREM2^+^ Macrophages ratio in anti-Csf1r and Isotype treatment condition (T-test. *P < 0.05, **P < 0.01, ***P < 0.001, ****P < 0.0001). (L-N) The difference in cell ratio of different treatment conditions (T-test. *P < 0.05, **P < 0.01, ***P < 0.001, ****P < 0.0001).

Furthermore, we explored the heterogeneity in macrophages, which consist of six subpopulations, among samples (Figure 3A). Among them, the TREM2^+^ subpopulation was characterized by high expression of GPNMB, TREM2, ACP5, LGMN, and TIMP2 (Figure 4A, Figure S3D), and mainly distributed at the boundary and core region of the tumor (Figure 3B, Figure S3E). Furthermore, we conducted flow cytometry on tumor and adjacent non-tumor tissues from HCC patients, and the results showed that the density of TREM2^+^ subpopulation was higher in tumor tissues (Figure S3H). Immunofluorescence staining further confirmed the presence of TREM2^+^ Macrophages in both human and HCC mouse models (Figure 3H). We also identified a subpopulation of macrophages with T cell characteristics, namely TCR^+^ Macrophages, characterized by high expression of IFITM1, FYN, NKG7, CD3E, GZMK, and GZMA (Figure S3D, Figure 4B). Furthermore, immunofluorescence staining confirmed the presence of this subpopulation in HCC tumor tissues (Figure 3G). To further investigate the robustness of these cell subpopulations, we divided the macrophages from the validation cohort into 10 subpopulations (Figure 3D), and reanalyzed the similarity of macrophages between the two independent cohorts using the anchor point algorithm proposed by Ramos et al. 28. Our findings suggested that the density of TREM2^+^ Macrophages subpopulation in our study cohort, as well as the expression patterns of TREM2 and SEPP1, were highly similar to those of the V_TREM2^+^ and V_SEPP1^+^macrophages subpopulations (Figure S3F). Furthermore, the similarity score was 0.59 between the TCR^+^ Macrophages subpopulation and the V_TCR^+^ Macrophages subpopulation, and 0.45 between the Macro^+^ Macrophages subpopulation and the V_Marco^+^ Macrophages subpopulation. These results further confirm the stable presence of TREM2^+^, MARCO^+^, and TCR^+^ Macrophages subpopulations (Figure 3E).

To investigate the relationship between these macrophage subpopulations and treatment responses, we conducted a statistical analysis of macrophage distribution in our study cohort. The proportion of TREM2^+^ Macrophages, at either the boundary or core region, was higher in non-responsive patients, compared to responsive patients (Figure 3C). In contrast, the proportion of TCR^+^ Macrophages within normal tissues showed no significant difference between responsive and non-responsive patients, but the proportion in the tumor boundary or core region was higher in responsive patients (Figure 3C). Consistently, both V_TREM2^+^ and V_SEPP1^+^ macrophages were more prevalent in non-responsive patients from the validation cohort. Notably, the V_SEPP1^+^ Macrophages subpopulation showed a statistically significant difference (Figure 3F, Figure S3G). Furthermore, results from other bulk RNA cohorts suggested that TREM2^+^ Macrophages were associated with a poor prognosis, while TCR^+^ Macrophages with a good overall survival (Figure S3I-L).

Functional characterization revealed that TREM2+ Macrophages were implicated in various immune-related processes, including chemotaxis, PD-1 signaling, and TGF-β production. Among all subsets, TREM2+ Macrophages exhibited the most potent angiogenic capabilities. In contrast, TCR^+^ Macrophages were enriched in terms of cytotoxicity, TCR signaling pathways, and positive regulation of T-cell activation, all associated with the inhibition of tumor progression (Figure S3M). These findings highlighted a functional divergence between distinct macrophage subsets. This conclusion was supported by Gene Ontology (GO) enrichment analysis of marker genes (Figure 4C-D). In the TCGA-LIHC cohort, we found a moderate-to-high correlation between TREM2 and all the immune checkpoints (Figure 4E). Additionally, spatial transcriptomic analysis of the two patients revealed a significant correlation between TREM2 expression and exhausted T cell signature (Figure 4E, Figure S4A-B), implying TREM2^+^ Macrophages as a significant contributor to limited efficacy of anti-PD-1 therapy.

Cellchat software revealed robust interactions specifically between TREM2^+^ Macrophages and C3_CD8^+^ Tex in tumor tissues, rather than in the border and normal tissues (Figure S4F).

These interactions included inhibitory ligand-receptor pairs, such as NECTIN2-TIGIT, LGALS9-HAVCR2, and CD86-CTLA4. Furthermore, TREM2^+^ Macrophages attracted exhausted CD8^+^ T cells through chemotaxis facilitated by CXCL12, thereby accelerating T cell exhaustion in tumor tissues (Figure S4C). Analysis of CXCL and CCL chemokine signaling pathways further demonstrated stronger immunoregulatory capabilities of TREM2^+^ Macrophages, compared to other types of macrophages (Figure S4G, Figure S4H). Additionally, a subpopulation of CD8^+^ T cells interacted with TCR^+^ Macrophages via the CD70 signaling pathway (Figure S4G), potentially enhancing the tumoricidal ability of these macrophages 30. In border and normal tissues, MARCO^+^ Macrophages (Kupffer cells) were also identified to promote T cell exhaustion through expressing molecules such as NECTIN2, PDCD1LG2, and LAGLS9, which is in agreement with previous studies (Figure S4D-E). Although exhibiting less pronounced impacts in these areas than in tumor tissues (Figure S4C-E), TREM2 on CD8^+^ T cells are still considered to play a broad immunosuppressive role.

We employed the CellphoneDB software to further investigate the cell types associated with TREM2^+^ Macrophages and exhausted CD8^+^ T cells within tumor tissues. Compared to other CD8^+^ T cells, CD8^+^ Tex cells exhibited a pronounced deficiency of inhibitory and stimulatory ligand-receptor pairs, when interacting with TREM2^+^ Macrophages in tumor tissues (Figure S4I). Exclusively, our analysis revealed a CSF1-CSF1R interaction between CD8^+^ Tex and TREM2^+^ Macrophages (Figure 4F), and the strength of this interaction was significantly higher than that observed in other macrophage subgroups (Figure 4F). Moreover, the inhibitory ligand-receptor pairs were predominantly concentrated within TREM2^+^ Macrophages and CD8^+^ Tex cells (Figure 4F). These findings suggested that a mutually reinforcing positive feedback loop exists between TREM2^+^ Macrophages and CD8^+^ Tex cells, and while receiving CSF1 from these T cell subsets, TREM2^+^ Macrophages provide sustained exhaustion signals to CD8^+^ Tex cells for proliferation, differentiation, and survival. This positive feedback loop may contribute to the resistance against anti-PD-1 therapy.

We conducted *in vivo* experiments to verify the efficacy of immunotherapy on TREM2^+^ Macrophages. A genetic HCC model was constructed using the Sleeping Beauty transposon cMyc-Nras system in *Trem2^-/-^* C57B/6J and wild-type (WT) mice, showing that the survival rate of *Trem2^-/-^* mice was better than that of WT mice (Figure 4G). In addition, we found that compared to WT mice, *Trem2^-/-^* mice had fewer tumors and lower liver-to-body weight ratios (Figure 4H). To study the effect of *Trem2* on the efficacy of anti-PD-1 therapy, we simultaneously administered anti-PD-1 therapy in *Trem2^-/-^* and WT mice, and the results showed that *Trem2^-/-^* mice were more responsive (Figure 4I). Since CSF1-CSF1R has a high specificity to TREM2^+^ Macrophages and CD8^+^ Tex cells, we explored the impacts of anti-PD-1 and anti-Csf1r treatment modalities on HCC in mice. Compared to isotype antibodies, both anti-PD-1 and anti-Csf1r treatments showed stronger effects, and their combination reaped the best outcomes (Figure 4J). We also found that anti-Csf1r therapy effectively suppressed the levels of *Trem2^+^* Macrophages in mice (Figure 4K, Figure S4J). Furthermore, anti-Csf1r therapy did not impact the overall abundance of CD8^+^ T cells (Figure 4L), but significantly diminished the population of PD-1^+^ CD8^+^ T cells and increased that of infiltrated NK cells, although these changes did not show statistical significance (Figure 4M-N). Based on these findings, we proposed that anti-Csf1r had the potential to suppress PD-1^+^ CD8^+^ T cell generation within the TME, and promote NK cell infiltration by reducing *Trem2^+^* Macrophages, thereby synergizing with anti-PD-1 therapy.

These findings suggest that TREM2^+^ Macrophages in HCC tumor tissues may impede the efficacy of anti-PD-1 therapy. However, this effect can be mitigated by inhibiting CSF1R to reduce the accumulation of TREM2^+^ Macrophages within the tumor tissue, thereby enhancing the effectiveness of anti-PD-1 therapy. Furthermore, it is hypothesized that TCR^+^ Macrophages within the tumor tissue exert an antitumor effect 29; however, this hypothesis should be further validated with more experimental data.

### IL1B^+^ cDC2s are the main executor on cDC2s

**Figure 5:**
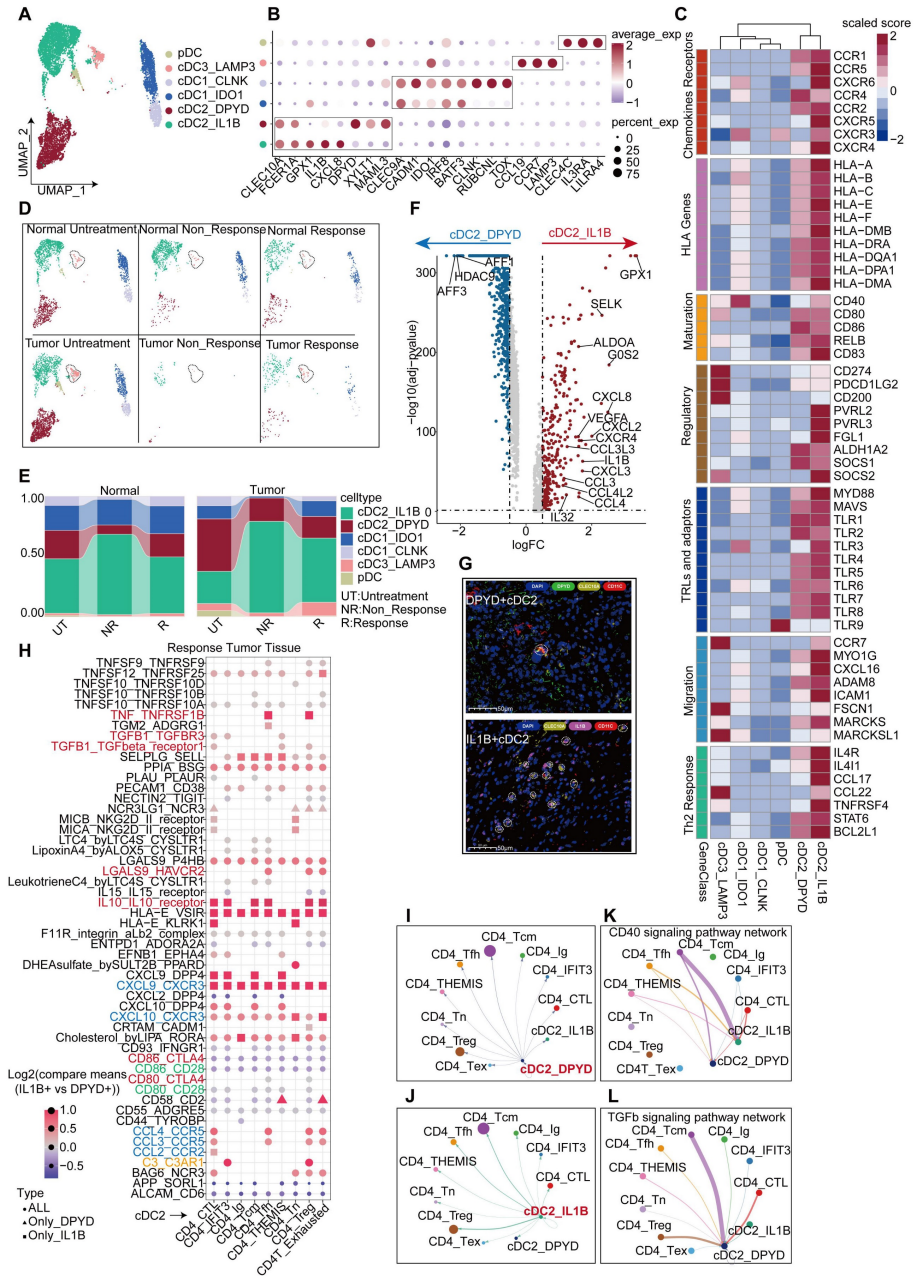
** IL1B^+^ cDC2s are the main executor on cDC2s.** UMAP plot showing DC subgroups of the discovery cohort. (B) Bubble plot displaying unique markers of different DC subgroups. (C) Differential expression of genes related to DC function across DC subgroups. (D) UMAP plot illustrating the distribution of DC subgroups across different tissues and under different treatment conditions. (E) Bar graph showing the proportion of DC subgroups across different tissues and under different treatment conditions. (F) Volcano plot depicting differential genes between two cDC2 subgroups. (G) Multicolor immunofluorescence staining confirms the presence of two types of cDC2 in human HCC tissues. (H) Bubble plot shows the differences in the co-receptor pairing between the two types of cDC2 and CD4^+^ T cell subgroups (p-values < 0.05, IL1B^+^ cDC2 vs DPYD^+^ cDC2). (I) Cell interactions between IL1B^+^ cDC2 dendritic cell and CD4^+^ T cell subgroups in the tumor tissue of non-responsive patients. (J) Cell interactions between DPYD^+^ cDC2 dendritic cell and CD4^+^ T cell subgroups in the tumor tissue of non-responsive patients. (K) Communication of the CD40 signaling pathway between cDC2 dendritic cells and CD4^+^ T cell subgroups. (L) Communication of the TGF-β signaling pathway between cDC2 dendritic cells and CD4^+^ T cell subgroups.

DCs act to support T cell proliferation and other functions, making the quantity and functionality of tumor-infiltrating DCs critical for the success of immunotherapy 30, 31, particularly in maintaining and expanding memory T cells 32. We collected DCs from HCC responders and non-responders to anti-PD-1 therapy, as well as non-treated individuals, and categorized them into two main groups. Within the total population of DCs, the proportion of plasmacytoid dendritic cells (pDCs), characterized by a high expression of CLEC4C and IL3RA 33, was found to be relatively low (Figure 5A, Figure 5E). The conventional dendritic cell (cDCs) subset comprises three major subtypes: traditional cDC1, cDC2, and cDC3 (mregDCs). Further analysis revealed that the cDC1 subset could be divided into two groups; one group expressing high levels of TOX, RUBCNL and CLNK, and one group lacking these characteristics but still expressing classical cDC1 markers (CELC9A and CADM1) (Figure 5A-B, Table S3). To validate the stability of these subpopulations, we isolated DCs from the validation cohort and divided them into five subpopulations (Figure S5B). Subsequent analysis revealed that the cDC2_c1 and cDC2_c2 subpopulations in the validation cohort exhibited a high similarity to the DPYD^+^ cDC2 and IL1B^+^ cDC2 subpopulations in the discovery cohort. pDC, cDC3_LAMP3, and cDC1_IDO1 subpopulations also displayed significant similarities in both cohorts (Figure S5C). Our findings continued to indicate a higher proportion of the IL1B^+^ cDC2 population in the tumor tissues of treatment-naïve patients, but not in their normal tissues (Figure 5D-E). Similar trends were also observed in the validation cohort, where the proportion of the cDC2_C1 population increased in the tumor tissues of treatment-naïve patients, compared to their normal tissues; however, statistical significance did not reach (Figure S5D). Conversely, no significant differences were found in the proportions of DPYD^+^ cDC2 and cDC2_C2 subpopulations between tumor and normal tissues (Figure 5E, Figure S5D). These results suggested a potential correlation between IL1B^+^ cDC2 abundance and treatment response. Furthermore, immunofluorescence staining of tumor tissues from HCC patients validated the characteristics of these two DC subpopulations (Figure 5G).

A comparative functional analysis of the two cDC2 subgroups revealed intriguing disparities. The IL1B^+^ cDC2 subgroup exhibited a greater functional prominence than the DPYD^+^ cDC2 subgroup, encompassing differential expression of chemokine receptors, HLA-D, TLRs, regulatory molecules, migratory capabilities, and support for Th2 cells (Figure 5C). IL1B^+^ cDC2s expressed higher abundances of chemokine ligands, receptors, and related cytokines (Figure 5F). GO enrichment analysis showed that upregulated genes in IL1B^+^ cDC2s were associated with enhanced chemotactic and migratory capabilities, as well as a regulatory role in T-cell differentiation (Figure S5F). Furthermore, a comparison in the validation cohort exhibited similar findings (Figure S5E). Survival analysis indicated that IL1B^+^ cDC2s appeared to exert a negative impact on the prognosis of patients (Figure S5G). Given that both IL1B^+^ cDC2 and DPYD^+^ cDC2 belong to the cDC2 subpopulation of DCs, we further observed a lower overall survival rate in patients with a higher level of IL1B^+^ cDC2 infiltration (Figure S5G-I).

We used Cellchat and CellPhoneDB to analyze the interaction between cDC2s and CD4^+^ T cells. The interaction between IL1B^+^ cDC2 and CD4^+^ T cells was more pronounced, particularly between CD4_Tcm and Treg cells (Figure 5I-J). Notably, IL1B^+^ cDC2 exhibited prominent expression of signaling molecules that stimulate DC activation, such as CD40-CD40L 34 (Figure 5K), whereas the CSF signaling pathway 35 was mainly activated in CD4_Tcm and Treg cells (Figure S5J). Conversely, DPYD^+^ cDC2 cells primarily displayed the stimulation of CD4_Tcm on the TGF-β signaling pathway, known to inhibit DC function and proliferation 36 (Figure 5L). To facilitate a more intuitive comparison of ligand-receptor pairs between the two cDC2 subgroups and CD4^+^T cells, we conducted a comparative analysis using CellphoneDB under identical conditions (Figure 5H). The results revealed that compared to DPYD^+^ cDC2IL1B^+^, cDC2 exhibited significantly higher expression levels of chemokine receptor pairs, particularly CXCL9-CXCR3 which is exclusively expressed by IL1B^+^ cDC2. Moreover, signaling pairs, such as TNF-TNFRSF1B, IL10-IL10R, LGALS9-HAVCR2, C3_C3AR1, and TGF-β1-TGF-βR1/3, displayed stronger interactions between IL1B^+^ cDC2 and CD4^+^ T cells, especially in Treg cells, the growth and development of which were significantly promoted. These findings partially elucidated why IL1B^+^ cDC2 is associated with an unfavorable prognosis in HCC patients and may represent one factor limiting the efficacy of anti-PD-1 therapy in this population. We must acknowledge that these data-driven analyses were insufficient to determine the overall contribution of the IL1B^+^ cDC2 subpopulation to TME immunity, as they merely reflect the intricate characteristics of the cDC2 subpopulation. Further comprehensive investigations are warranted to elucidate the roles of this subpopulation.

### Transcriptional changes of CD8^+^ T cells after anti-PD-1 treatment

**Figure 6:**
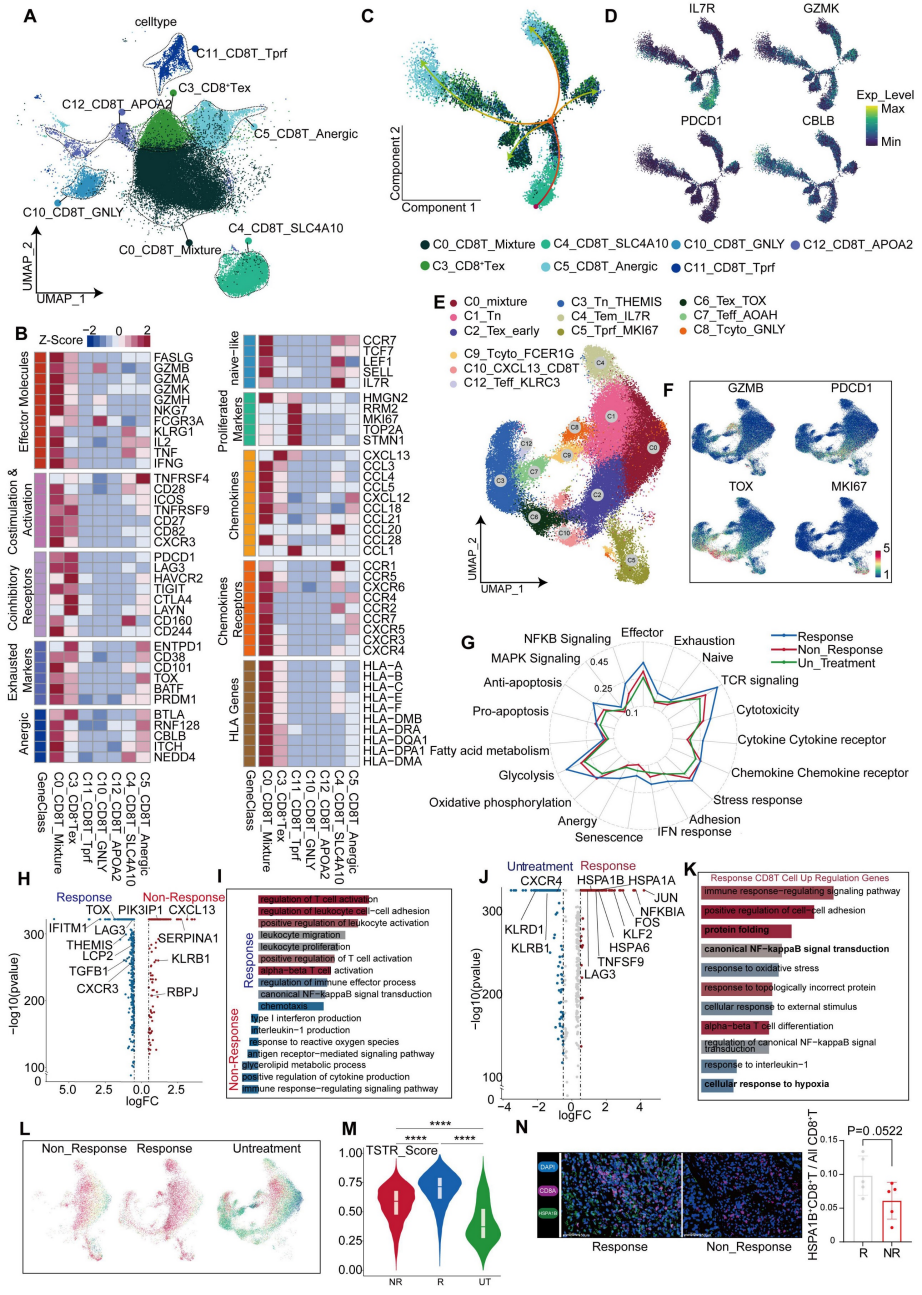
** Transcriptional changes of CD8^+^ T cells after anti-PD-1 treatment.** (A) UMAP plot showing CD8^+^ T cell subgroups. (B) Differential expression of genes related to CD8^+^ T cell function across subgroups. (C) CD8^+^ T cell developmental trajectory simulated with monocle2. (D) IL7R, GZMK, PDCD1, and CBLB expression levels along the developmental trajectory. (E) UMAP plot for re-clustering and defining CD8^+^ T cells from the discovery cohort. (F) Feature plot showing expression levels of GZMB, PDCD1, TOX, and MKI67. (G) Gene Set Enrichment Index of all CD8^+^ T cells from responsive, non-responsive, and untreated patients. (H) Volcano plot showing differential genes in CD8^+^ T cells from responsive and non-responsive patients. (I) GO enrichment of differential genes between responsive and non-responsive groups. (J) Volcano plot showing differential genes in CD8^+^ T cells from responsive and untreated patients. (K) GO enrichment of up-regulated differential genes in CD8^+^ T cells from responsive patients. (L) Scoring of the TSTR signature in CD8^+^ T cells from non-responsive, responsive, and untreated patients. (M) Violin and box plots showing TSTR scores in CD8^+^ T cells from responsive, non-responsive, and untreated groups (Wilcoxon test. *P < 0.05, **P < 0.01, ***P < 0.001, ****P < 0.0001). (N) Multicolor immunofluorescence shows a statistically significant difference in the abundance of HSPA1B^+^ CD8^+^ T cells between responders and non-responders in the treatment (T-test. *P < 0.05, **P < 0.01, ***P < 0.001, ****P < 0.0001).

After a comprehensive analysis, all CD8^+^ T cells were divided into seven distinct subgroups (Figure 6A), including a proliferative subgroup characterized by high expression of MKI67 and TOP2A, an exhausted subgroup of CD8^+^ T cells by high expression of PDCD1, LAG3, HAVCR2, and CTLA4, and a subgroup by high expression of GNLY 37, indicative of a predisposition towards cytotoxic T cells.

Additionally, a subgroup expressing high levels of NEAT1^38^ and LRBA 39 was identified as a marker typically associated with T cell dysfunction 40 (Figure S1H). The C0 cluster exhibited diverse cellular characteristics; therefore, it was termed as a mixed CD8^+^ T cell population (Figure 6B). Gene set enrichment analysis (GSEA) further teased out distinct phenotypic traits in each subgroup (Figure S6C, Figure S6E). All CD8^+^ T subgroups were found in the tissues of different regions, without significant statistical differences in their proportions (Figure S6A-B). A notable reduction was observed in CD8^+^ T cells in tumor tissues from non-responsive patients, despite inequality between sizes of responsive and non-responsive patients (responsive: non-responsive = 6:1), suggesting that the reduction in CD8^+^ T cells keeps significant regardless of the difference between proportions of patients. This characteristic was also observed in border tissues, but absent in normal tissues (Figure S6D), implying a link between immunotherapeutic outcomes and the abundance of CD8^+^ T cells within both tumor core and border tissues.

To gain deeper insights into the effects of CD8^+^ T cells on the outcomes of anti-PD-1 therapy, we combined the data of CD8^+^ T cells from the tumor tissues of untreated and non-responsive patients (Figure S6F-G). Furthermore, we incorporated an additional validation cohort comprising patients with treatment responses and non-responses, as well as untreated patients. CD8^+^ T cells were isolated from their tumor tissues for subpopulation analysis (Figure S8A), and further categorized into 12 subgroups based on classical markers and common attributes of CD8^+^ T cells, including naïve, effector, cytotoxicity, exhaustion, and senescence (Figure 6E-F, Figure S7B, Table S4).

**Figure 7:**
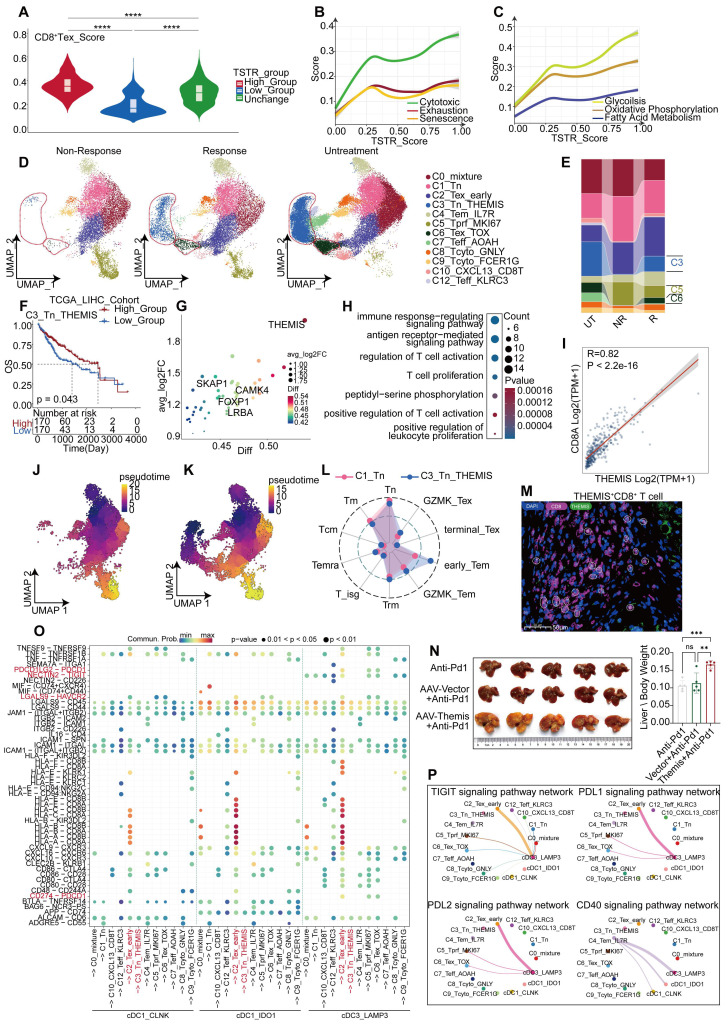
** Transcriptional changes of CD8^+^T cells after anti-PD-1 treatment.** (A) Grouping of CD8^+^ T cells based on TSTR scores, shown with box and violin plots for exhaustion scores (Wilcoxon test. *P < 0.05, **P < 0.01, ***P < 0.001, ****P < 0.0001). (B) Trends in cytotoxic, exhaustion, and senescence scores about TSTR scores. (C) Trends in glucose metabolism, oxidative phosphorylation, and fatty acid metabolism about TSTR scores. (D) UMAP plot showing the distribution of CD8^+^ T cell subgroup in responsive, non-responsive, and untreated patients. (E) Bar graph showing the proportion of CD8^+^ T cell subgroup in responsive, non-responsive, and untreated patients. (F) Kaplan-Meier survival analysis of the signature of C3_Tn_THEMIS in the TCGA-LIHC cohort. (G) Bubble plot showing Top30 marker genes of C3_Tn_THEMIS. (H) GO enrichment results for marker genes of C3_Tn_THEMIS. (I) Correlation of CD8A and THEMIS in the TCGA-LIHC cohort. (J) UMAP plot illustrating the developmental trajectory of non-responsive CD8^+^ T cells simulated with monocle3. (K) UMAP plot showing the developmental trajectory of responsive CD8^+^ T cells simulated with monocle3. (L) Radar plot showing cell type inclination scores for C1_Tn and C3_Tn_THEMIS subgroups. (M) Multicolor immunofluorescence staining confirms the presence of THEMIS^+^ CD8^+^ T cells in human HCC tissues. (N) tumor characteristics of HCC-mouse models after treatment with anti-PD-1 and anti-PD-1 combined with AAV-Themis (T-test. *P < 0.05, **P < 0.01, ***P < 0.001, ****P < 0.0001). (O) Bubble plot showing cell communication between cDC1, LAMP3^+^ cDC3, and various types of CD8^+^ T cells in the tumor tissue of responsive patients. (P) Communication of TIGIT, PDL1, PDL2, and CD40 signaling pathways between cDC1 and CD8^+^ T cells.

A comprehensive analysis was conducted on CD8^+^ T cells from untreated, responsive, and non-responsive patients. CD8^+^ T cells from responsive patients exhibited more pronounced glucose metabolism and TCR signaling than untreated CD8^+^ T cells (Figure 6G, Figure S8B). Contrary to our expectations, CD8^+^ T cells from responsive patients exhibited a greater degree of exhaustion, including elevated TOX and LAG3 expression (Figure 6H). However, genes associated with CD8^+^ T cell differentiation, such as THEMIS 42, and genes maintaining IFN-γ levels, such as IFITM1, were found at higher expression levels (Figure 6H). Functionally, terms related to CD8^+^ T cell activation were enriched in responsive patients, because of the effects of anti-PD-1 therapy (Figure 6I). Using pan-cancer analyses 43, Chu et al. have identified a subset of stressed CD8^+^ T cells characterized by elevated expression of HSP family genes, JUN, FOS, and NFKBIA; this subset was also observed in our comparative analysis between responsive and untreated patients (Figure 6J). Functions of these cells included protein folding, hypoxia response (Figure 6K, Figure S8E), and activation of NF-κB pathways (Figure 6K), which is consistent with Chu's descriptions 43. To validate our findings in the gene sets provided by Chu et al., we employed the AUcell algorithm for single-cell scoring, yielding results consistent with our expectations. The highest score was observed in responsive patients, followed by non-responsive patients, and the lowest in untreated patients (Figure 6L-M, Figure S8G). Additionally, the expression levels of stress-related gene HSPA1B were consistent with those observed across the three groups (Figure S7C) and confirmed by mIHC analysis (Figure 6N). We further revealed a positive correlation between stress level and exhaustion score (Figure 7A). Similarly, we discovered that elevated stress levels generally corresponded to enhancements in CD8^+^ T cell cytotoxic activity, exhaustion, and senescence (Figure 7B, Figure S8H). Despite what we found in the validation cohort, both the cytotoxicity and exhaustion of CD8^+^T cells demonstrated a decreasing trend during the early phase of TSTR (Figure S8H), with cytotoxicity more associated with stress than exhaustion alone (Figure 7B, Figure S8H). Furthermore, a higher level of stress was linked to a gradual enhancement of glucose metabolism, fatty acid metabolism, and oxidative phosphorylation in CD8^+^ T cells (Figure 7C, Figure S8I), suggesting that elevated stress levels indicate hyperactivity of CD8^+^ T cells. Responsive patients exhibited higher stress levels, potentially due to the reactivation of T-cells following anti-PD-1 therapy. Non-responsive patients showed a low tumor-killing ability of CD8^+^ T cells, but it does not imply that CD8^+^ T cells take no role in immune responses to blockade treatment.

A significant decrease in C6_Tex_TOX and C3_Tn_THEMIS subgroups was observed in non-responsive patients compared to responsive patients, accompanied by an increase in the C5_Tprf_MKI67 subgroup (Figures 7D-E). We confirmed the presence of THEMIS^+^ CD8^+^ T cells using immunofluorescence staining (Figure 7M). The C3 subgroup with high expression of THEMIS 42 and SKAP1 44 presented T cell proliferation and activation (Figure 7G), along with elevated glucose metabolism and TCR signaling scores (Figure 7H). Moreover, the survival rate increased in patients exhibiting a higher level of the C3 subgroup (Figure 7F), while the C6 subgroup exhibited high expression of TOX and PDCD1 (Figure S7E).

The C5_Tprf_MKI67 subgroup was characterized by high expression of TOP2A, MKI67, and HMGB2 (Figure S7F), as well as enhancements of glucose metabolism and oxidative phosphorylation (Figure S7H), and predominantly associated with cell proliferation (Figure S7G). Cell trajectory analysis based on Tn and Naïve scoring (Figure S7B) identified both C3 and C1 subgroups as potential developmental starting points for CD8^+^ T cells (Figure 7J-K), wherein cell type propensity analysis revealed high naïve scores of both subgroups. However, C3_Tn_THEMIS displayed more pronounced characteristics of early-stage Tem, relative to C1_Tn (Figure 7L), suggesting that C3_Tn_THEMIS may represent an immature state of Tem capable of further expansion and functional engagement. Additionally, C6_Tex_TOX displayed developmental features closer to C3_Tn_THEMIS than to C1_Tn (Figure 7K), suggesting that C6_Tex_TOX might originate from C3_Tn_THEMIS, which was conspicuously absent in non-responsive patients (Figure 7J). C3_Tn_THEMIS in the tumor tissues of non-responsive patients may account for the lack of response to anti-PD-1 therapy. THEMIS plays a crucial role in T-cell differentiation, and we observed high expression of THEMIS in C3_Tn_THEMIS cells. Furthermore, THEMIS levels decreased after PD-1 treatment in the discovery and validation cohorts, with non-responders showing lower THEMIS levels than others (Figure S8J-M). In addition, genes highly correlated with THEMIS might regulate the cytotoxicity and activity of DCs (CLEC9A, CD40LG), naïve T cells (CCR7, SELL), and CD8^+^ T cell (CD8A, GZMK, LCK 45) in the TCGA-LIHC cohort (Figure S7J). These findings suggested that THEMIS modulation might tune the efficacy of anti-PD-1 therapy on T cell responses. Consequently, we reduced the expression of THEMIS in mice by injecting AAV-Themis intravenously during the administration of anti-PD-1 therapy. The results showed that the effectiveness of anti-PD-1 therapy was significantly reduced after the intervention of AAV-Themis, implying a contribution by THEMS to the limited efficacy of anti-PD-1 therapy (Figure 7N).

Analysis of cellular interactions revealed that C3_Tn_THEMIS exhibited the weakest interactions with other CD8^+^T cells, whereas C2_Tex_early displayed the strongest interactions (Figure S7K). Notably, the inhibitory ligand-receptor interactions between LAMP3^+^ cDC3 and CD8^+^ T cells were more pronounced in the other two cDC1 subgroups (Figure 7O-P), suggesting a potential role of LAMP3^+^ cDC3 in regulating CD8^+^ T cell exhaustion. Additionally, C4_Tem_IL7R was identified as the primary cell cluster promoting cDC1 activation via CD40 signaling (Figure 7P). Although these findings may not directly correlate with responses or non-responses to immunotherapy, they provide a new prospective into the immune TME during anti-PD-1 treatment.

### Transcriptional changes of CD4^+^ T cells after anti-PD-1 treatment

**Figure 8:**
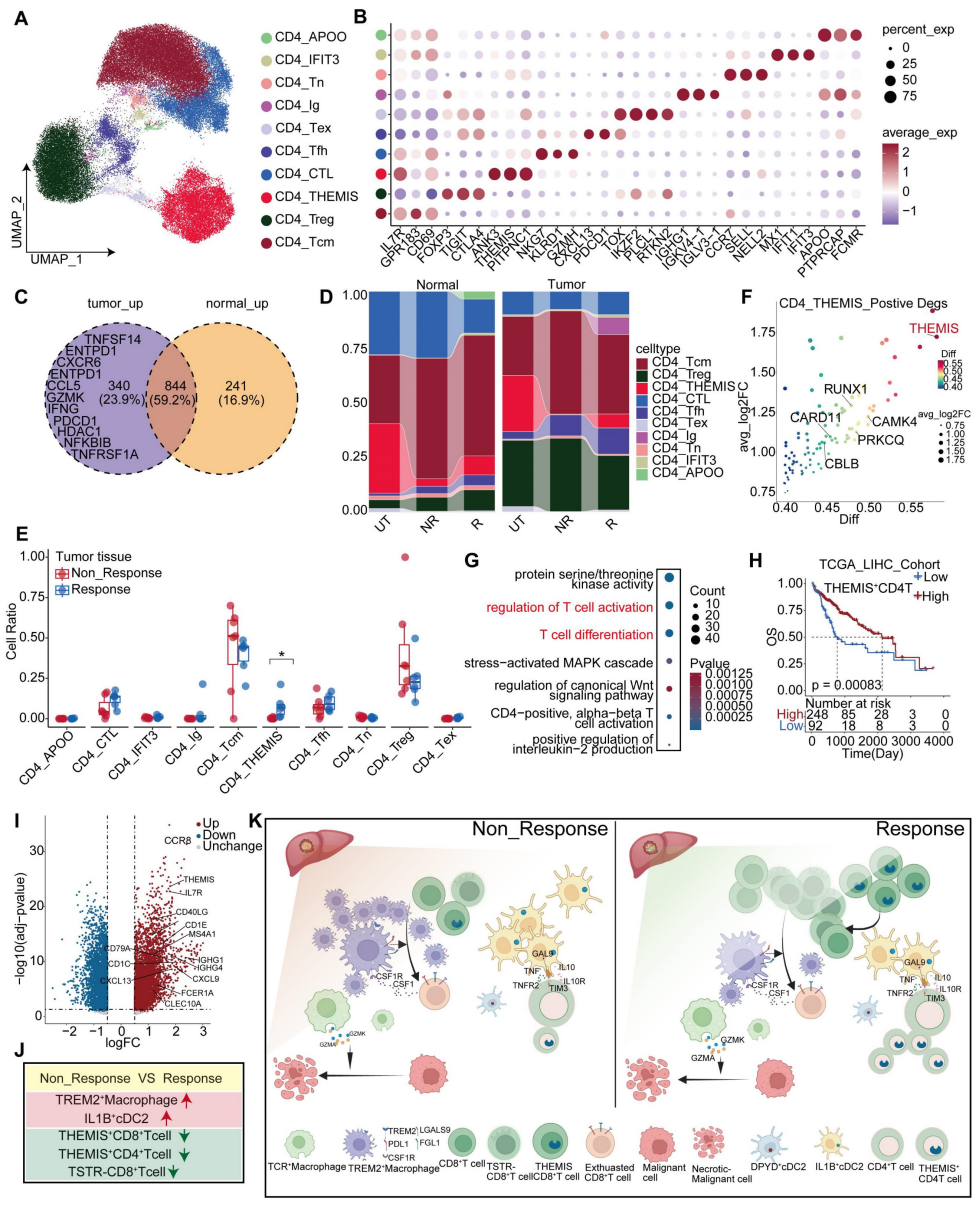
** Transcriptional changes of CD4^+^T cells after anti-PD-1 treatment.** (A) The UMAP plot illustrates the subpopulations of CD4^+^ T cells in the discovery cohort. (B) The marker genes associated with CD4^+^ T cell subpopulations are identified in the discovery cohort. (C) Comparison of differentially expressed genes in CD4^+^ T cells between responders and non-responders to anti-PD-1 treatment in tumor and normal tissues. (D) The figure shows the distribution of CD4^+^ T cell subpopulations in the discovery cohort across different tissues and treatment conditions. (E) The distribution of CD4^+^ T cell subpopulations in tumor tissue across responders and non-responders in the discovery cohort. (Wilcoxon test. *P < 0.05, **P < 0.01, ***P < 0.001, ****P < 0.0001). (F) The bubble plot shows the top 50 genes of the CD4_THEMIS subpopulation. (G) GO enrichment results for marker genes of CD4_THEMIS. (H) Kaplan-Meier survival analysis of the signature of CD4_THEMIS in the TCGA-LIHC cohort. (I) The differentially expressed genes between the high and low infiltration levels of CD4_THEMIS in TCGA-LIHC. (J-K) The legend illustrates the main findings of this study.

Multiple datasets were integrated to compare the profiles of CD4^+^ T cells between untreated, responsive, and non-responsive patients. We identified 10 clusters of CD4^+^ T cells (Figure 8A, Table S5), including well-known subsets such as regulatory T cells (Tregs) expressing FOXP3 46, naïve CD4^+^ T cells (Tn) expressing CCR7 and SELL 41, and exhausted CD4^+^ T cells (Tex) expressing TOX 47. Moreover, we discovered a sub-cluster expressing GZMH and NKG7, two cytotoxic markers associated with CD8^+^ T cells; this sub-cluster was named CD4_CTL 48. Another cluster exhibiting high expression of IL7R, GPR183, and CD69 was designated as CD4_Tcm 41, while other clusters showed elevated THEMIS expression (Figure 8B, Figure S9I). Relative to responsive patients, non-responsive patients revealed a low specificity to normal or tumor tissues in the distribution of differentially expressed genes (DEGs) within the CD4^+^ T cell population. Specifically, 340 genes were upregulated in tumor tissues compared to normal tissues, but most were overlapped between the two groups (Figure 8C). These tumor-specific upregulated genes included inhibitory receptors, such as ENTPD-1 49 and PDCD1, as well as effector molecules, such as GZMK and IFNG, all involved in antitumor activity. Additionally, we further characterized CD4^+^ T cell populations in the responders and non-responders of the validation cohort, respectively (Figure S10A-D).

Both Treg cells and CD4_Tfh cells increased significantly in tumor tissues. In contrast, CD4_CTL cells were more prevalent in normal tissues than in tumor tissues (Figure S9A-B). Furthermore, the proportion of CD4_CTL cells was higher than that in non-responsive patients (Figure S9C), which is consistent with Ramanuj's study 50. Notably, the CD4_THEMIS subgroup was the only group that showed a significant between-group difference, and was almost absent in the tumor tissues of non-responsive patients (Figure 8E). Similarly, the C4 subgroup was identified in the tumor tissues of the validation cohort, exhibiting a high similarity to CD4_THEMIS cells and expressing high levels of CBLB, THEMIS, and CAMK4 (Figure S10F). This subgroup was also significantly abundant in responsive patients, but not observed in normal tissues (Figure S10C-E).

Moreover, CD4_THEMIS expressed high levels of THEMIS and other genes, such as RUNX1 51 and CAMK4 52, which promote T cell proliferation and activation (Figure 8F). Our propensity score analysis demonstrated that CD4_THEMIS cells were closer to Tcm cells (Figure S9D). Functional analysis further demonstrated that CD4_THEMIS cells exhibited similar functions to those of CD8_THEMIS cells, including T cell activation and proliferation (Figure 8G). Importantly, previous research has suggested an essential role of THEMIS in T cell function, and we found that THEMIS was highly and specifically expressed in T cells (Figure S9H), indicating a good prognosis across two independent cohorts (Figure S9F-G). Upon stratifying patients in the TCGA-LIHC cohort into high and low groups based on THEMIS^+^CD4^+^T cell infiltration, differential and functional analyses revealed that elevated expression of molecules was associated with DCs (CLEC10A, CD1C, FCER1A, and CD40LG), B cells, and plasma cells (CD79A, MS4A1, and IGHG1), as well as various chemokines and chemokine receptors (Figure 8I). These molecules are involved in antigen presentation, B-cell-related pathways, and chemotaxis. These suggest that this cell type has the potential to regulate the immune system through multiple mechanisms (Figure S9E).

Next, we observed that TREM2^+^ Macrophages exhibited high MHC-II expression and antigen presentation (Figure S3M), which sparked our interest in investigating the crosstalk between CD4^+^ T cells and macrophages. Cellchat analysis revealed that TCR^+^ Macrophages displayed the closest interaction with CD4^+^ T cells in tumor tissues from responsive patients, whereas TREM2^+^ Macrophages were predominantly associated with CD4^+^ T cells in tumor tissues from non-responsive patients (Figure S9J). We identified ligand-receptor pairs involved in the interactions between CD4^+^ T cells and macrophages, specifically in the tumor tissues of non-responsive patients. Chemokines and their corresponding receptors were primarily found on TREM2^+^ Macrophages and CD4^+^ T cells, where TREM2^+^ Macrophages inhibited CD4^+^ T cell function through multiple immunosuppressive ligands (Figure S10H), including PDL2 signals specifically targeting CD4_Tfh (Figure S9M). Conversely, TGF-β signaling mainly affected TREM2^+^ Macrophages (Figure S9L), reprogramming them into a pro-tumoral phenotype characterized by angiogenesis and maintenance of an immunosuppressive microenvironment. Interestingly, IL-2 53, crucial for promoting the proliferation and differentiation of Tregs, was predominantly stimulated by CD4 ^+^ Tfh cells, potentially representing a key regulatory mechanism controlling Treg activity (Figure S10G).

In summary, we observed elevated levels of THEMIS^+^ CD4^+^ T cells in tumor tissues from non-responsive patients, suggesting that this cell population may modulate the efficacy of anti-PD-1 treatment by regulating CD4^+^ T cell development. Additionally, the interaction between TCR^+^ Macrophages and CD4^+^ T cells was most pronounced in tumor tissues from responsive patients, whereas that between TREM2^+^ Macrophages and CD4^+^ T cells was more intimate in tumor tissues from non-responsive patients. Notably, TREM2^+^ Macrophages promoted the transition of CD4^+^ T cells into an anti-tumor phenotype through inhibitory receptors such as PDL2 and other inhibitory receptors, while CD4_Tcm further enhanced the differentiation of TREM2^+^ Macrophages into a tumor-promoting phenotype via the TGF-β pathway.

## Discussion

The efficacy of immunotherapy on HCC is influenced by several factors, including mutational burden 54, lack of immune cells (cold tumor) 55, an immunosuppressive TME, and barriers impeding T cell infiltration around the tumor 16. Complex crosstalk among players in the immunosuppressive microenvironment is a key factor 55. Here, through a comprehensive analysis of responders and non-responders to PD-1 treatment, along with myeloid and T-cell subpopulations in untreated patients, we identified previously unreported features of the TME in HCC patients (Figure 8J-K). We discovered that not all patients with immune barriers exhibited poor treatment outcomes, which were largely dependent on CD8^+^T cell infiltration levels. TREM2^+^ and TCR^+^ Macrophages may play contradictory roles in the HCC TME. In non-responsive patients, the proportion of TREM2^+^ Macrophages increased. Previous studies have also indicated an inhibitory role of TREM2^+^ Macrophages in various tumors 56. However, the precise role of TCR^+^ Macrophages in HCC remains unclear.

Our research revealed that TREM2^+^ Macrophages can directly induce CD8^+^ T cell exhaustion through ligand-receptor interactions, such as NECTIN2-TIGIT/LGALS9-HAVCR2, and recruit a substantial number of CD8^+^ T cells via CXCL and CCL-related chemokines, thereby exacerbating their exhaustion. anti-Csf1r effectively hinders the accumulation of Trem2^+^ Macrophages in HCC tumor tissues and synergistically enhances the therapeutic efficacy of anti-PD-1. Conversely, TCR^+^ Macrophages can directly eliminate tumor cells using cytotoxic granules, such as GZMA and GZMK. The cDC2 population can be functionally classified into two subgroups, with the IL1B^+^cDC2 subgroup undertaking the primary role. The CD40-CD40L co-receptor system is essential for DC activation, primarily mediated by CD4_Tcm cells; however, it exerts a significantly stronger stimulus on IL1B^+^ cDC2 than on DPYD^+^ cDC2. Moreover, IL1B^+^ cDC2 highly expresses NECTIN2 which mediates the suppression of CD4^+^ T cells via either CD226 57 or TIGIT. Targeted blockade of NECTIN2 may enhance the intra-tumoral activation of CD4^+^ T cells.

It has been demonstrated that CD8^+^ T cells can induce the expression of PD-1 through endoplasmic reticulum stress 58. Here, we found that CD8^+^ T cells also fall into a heightened state of stress in non-responsive patients, which may be associated with the transition of T cells into a full-play mode following treatment with anti-PD-1 inhibitors. In this state, the cytotoxic activity of T-cells is enhanced, and their exhaustion and senescence accelerated. We observed elevated stress levels in CD8^+^ T cells in PD-1-responsive patients. Further investigation is required to determine whether this state contributes to the acceleration of CD8^+^ T cell exhaustion or senescence. Additionally, we identified a subset of CD8^+^ T cells expressing a high level of THEMIS but absent in non-responsive patients. We hypothesize that these cells have the capacity to further proliferate and eventual differentiate into terminally exhausted T-cells characterized by high levels of PD-1 and CTLA4 expression, and their absence may contribute to the poor outcomes of immunotherapy in non-responsive patients. Additional analyses using TCR sequencing provided insights into the function of these tumor-reactive T cells. Similarly, we observed the absence of CD4^+^ T cells with high THEMIS expression in non-responsive patients. Furthermore, inhibition on THEMIS expression may discount the efficacy of anti-PD-1 therapy. The AAV-Themis we used did not bind specifically to CD8 or CD4 promoters, which resulted in a weaker suppression on T cells by the AAV virus. Therefore, we cannot conclude by announcing that THEMIS acts by relying on T cells, or a specific subpopulation of T cells in combination with PD-1. It remains unclear how THEMIS regulates T-cell function, and its negative impact on anti-PD-1 therapy efficacy should be addressed in future investigations.

In non-responsive patients, TREM2^+^ macrophages and CD4^+^ T cells exhibited more intimate interactions. Numerous CD4^+^ T cells reprogram TREM2^+^ macrophages by means of TGF-β. Additional CD4 ^+^ Tfh cells promote the differentiation of Treg cells via IL2 59, thereby fostering an immunosuppressive TME. Other factors in the TME of HCC wait to be explored. For instance, neutrophils can tilt the balance within the TME through various mechanisms, and specific monocyte subgroups may charge the functionality of CD8^+^ T cells through cytokine signaling. As primary components of tumor tissue, tumor cells can suppress an immune TME through various mechanisms, such as PDL1 expression on their cell membrane and release of immunosuppressive molecules like TGF-β 60.

This study investigated the factors accounting for the poor responses to anti-PD-1 therapy in HCC patients; however, some of our findings through data analysis require validation through more rigorous cell or animal experiments, as well as large-scale clinical samples. Nonetheless, the cells and molecules, which we found to present unique functions in this study, may be targeted to improve the efficacy of immunotherapies for HCC.

## Supplementary Material

Supplementary figures.

Supplementary tables.

## Abbreviations

HCC: hepatocellular carcinoma
PD-1: programmed cell death-1
ORR: objective response rates
TME: tumor microenvironment
cDC1: type 1 dendritic cell
cDC2: type 2 dendritic cell
pDC: plasmacytoid dendritic cell
TAM: tumor-associated macrophage
CAF: cancer-associated fibroblast
Treg: regulatory T cell
IMC: imaging mass cytometry
TREM2: Triggering receptor expressed on myeloid cell 2
TCGA: The Cancer Genome Atla
WT: wild-type
Tcm: central memory T cell
mIHC: Multiplex Fluorescence Immunohistochemistry Technique
Tem: effector memory T cell
CTL: Cytotoxic T lymphocyte
TGF-β: Transforming growth factor β
