# Supplementary material for: Single cell analyses reveal the PD-1 blockade response-related immune features in hepatocellular carcinoma
Source: Theranostics. 2024 Jun 1;14(9):3526–47. doi: 10.7150/thno.95971 (PMC11209711; doi:10.7150/thno.95971)
Supplement: Supplementary file 1 — Supplementary figures. [file thnov14p3526s1.pdf]

Supplementary Figures

Figure S1

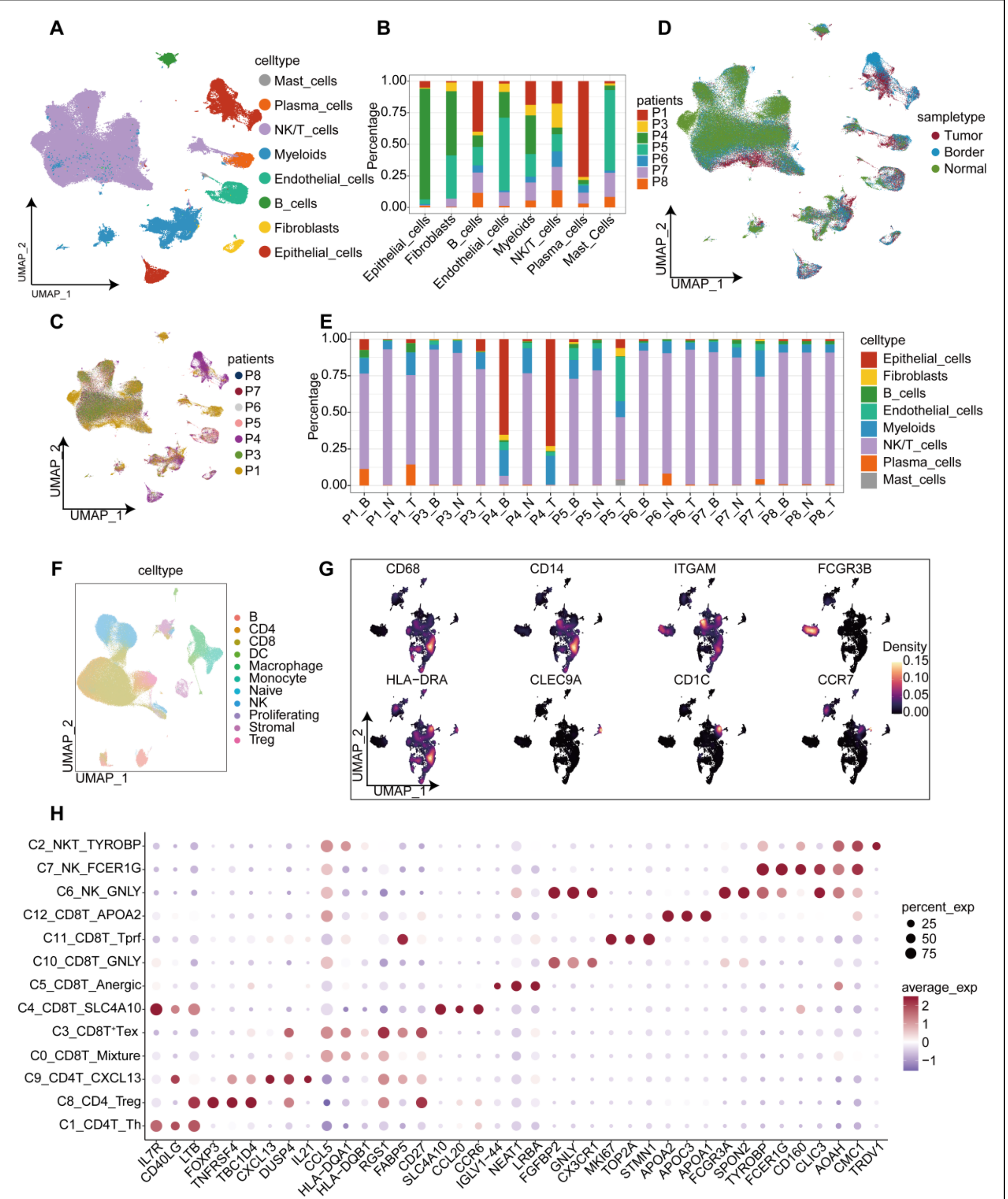

Figure S1. Single cell atlas of HCC samples responsive and non-responsive to PD-1 blockade.

(A) Cell type maps for this study cohort. (B) Bar graph illustrating the proportion of cell types in different tissue sites for each patient. (C) UMAP plot displaying the patient-type origin of cells. (D) UMAP plot showing the tissue type origin of cells. (E) Bar graph showing the composition ratio of each cell type across patients. (F) Cell type maps for GSE206325 cohort. (G) UMAP plot illustrating markers of myeloid cell subpopulations. (H) Bubble plot displaying unique markers of NK/T cell subpopulations.

Figure S2

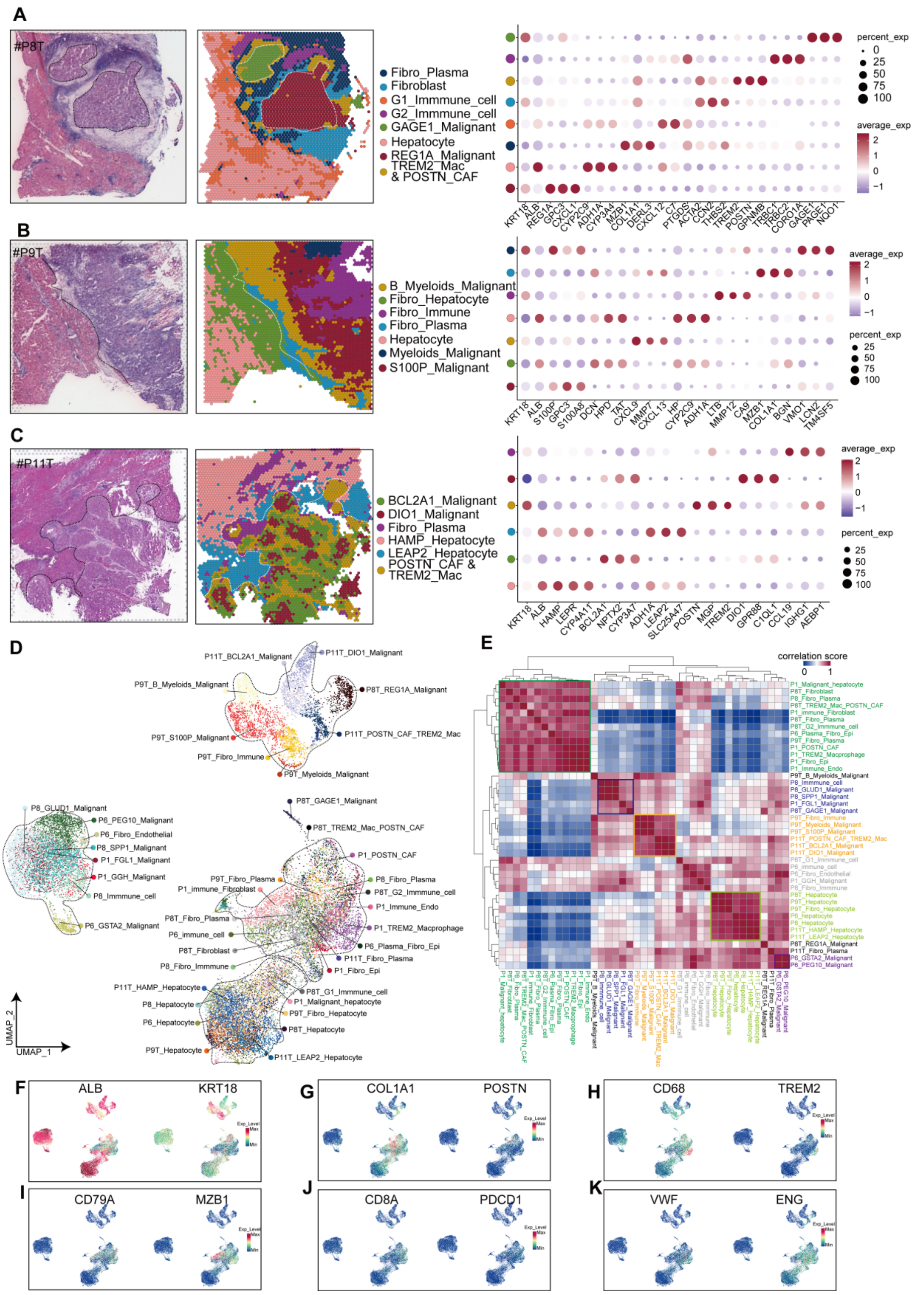

Figure S2. Spatial transcriptomic features of responsive and non-responsive HCC adjacent tissues.

(A-C) Cell types and corresponding markers in patients. (D) UMAP plot of all spots and their corresponding cell types. (E) Correlation between cell subgroups. (F-K) UMAP plots show the expression of select markers across all spots.

Figure S3

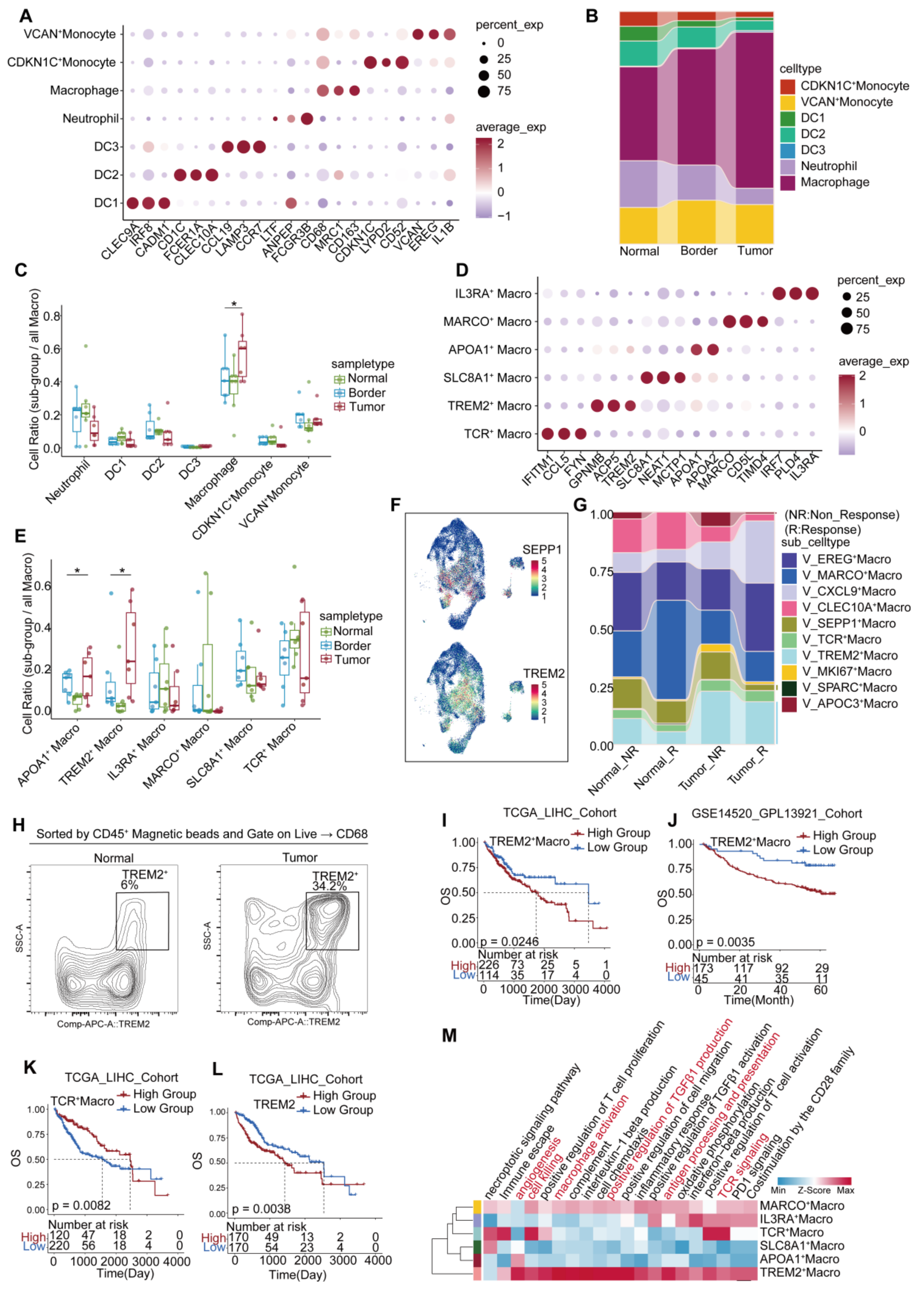

Figure S3. TREM2+ Macrophages represent a predominant immunosuppressive subset within the macrophage population.

(A) Bubble plot showing markers corresponding to myeloid cells subgroup. (B) Bar graph illustrating the proportion of myeloid cells subgroup across tissues. (C) Proportion statistics of myeloid cells subgroup for individual patients across different tissues (Wilcoxon test. *P < 0.05, **P < 0.01, ***P < 0.001, ****P < 0.0001). (D) Bubble plot showing markers

corresponding to Macrophage subgroups. (E) Proportion statistics of Macrophage subgroups for individual patients across different tissues (Wilcoxon test. $*P < 0.05$, $**P < 0.01$, $***P < 0.001$, $****P < 0.0001$). (F) UAMP plot showing SEPP1 and TREM2 expression level of macrophages in GSE206325 cohort. (G) Bar graph illustrating the proportion of macrophage subgroup across tissue and treated type in GSE206325 cohort. (H) Quantification of $TREM2^+$ macrophages in normal and tumor tissue of HCC patient by flow cytometry. (I) Kaplan-Meier survival analysis of $TREM2^+$ macrophage signature in the TCGA-LIHC cohort. (J) Kaplan-Meier survival analysis of $TREM2^+$ macrophage signature in the GSE14520 cohort. (K) Kaplan-Meier survival analysis of TCR^+ macrophage signature in the TCGA-LIHC cohort. (L) Kaplan-Meier survival analysis of TREM2 in the TCGA-LIHC cohort. (M) Heatmap representation of functional characteristics of macrophage subgroups, based on single-sample gene set enrichment analysis (ssGSEA) scores, normalized across macrophage subgroups.

Figure S4

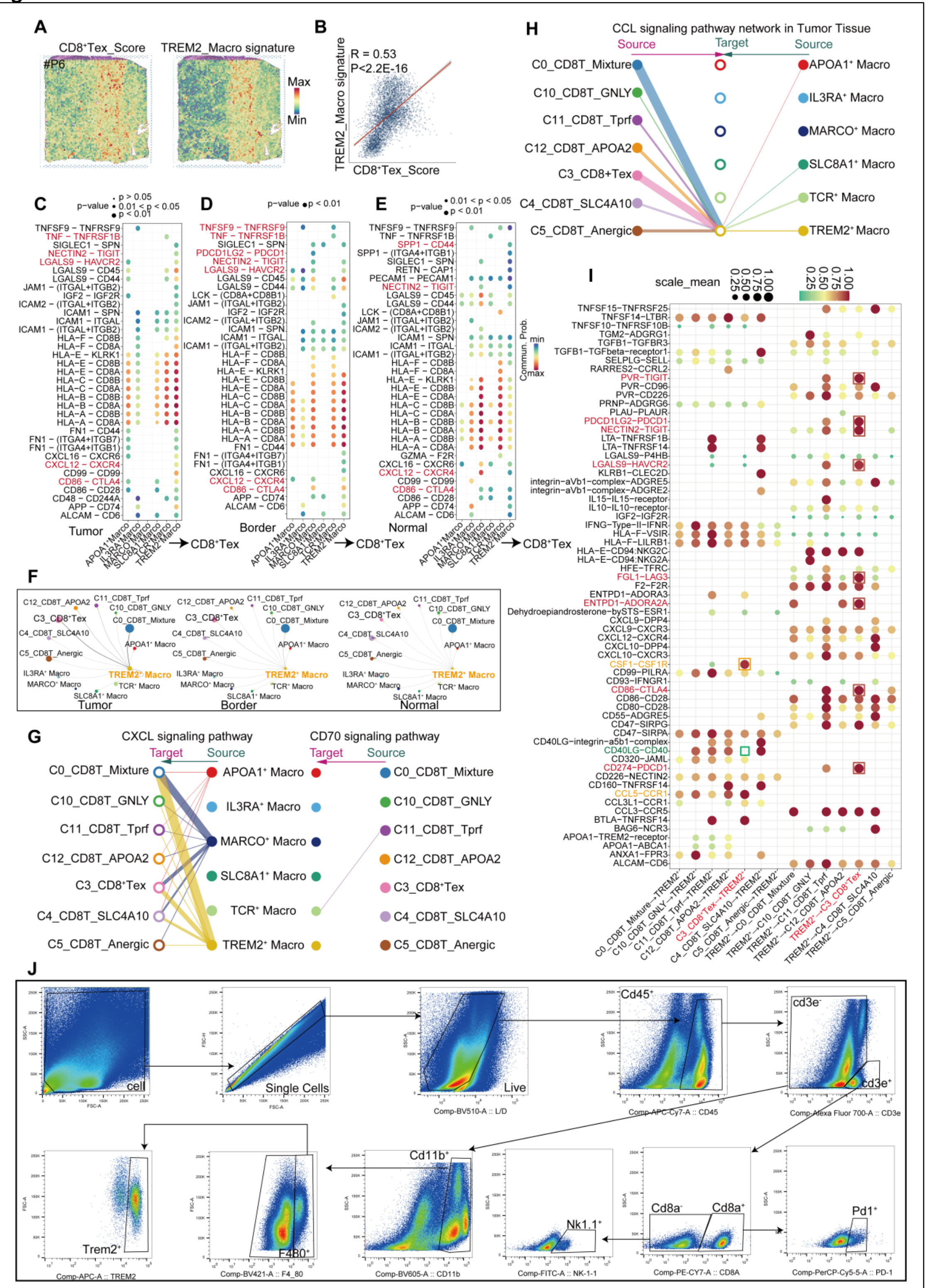

Figure S4. TREM2$^+$ Macrophages represent a predominant immunosuppressive subset within the macrophage population.

(A-B) Correlation between TREM2$^+$ macrophage signature and CD8$^+$ Tex signature in P6. (C-E) Ligand-receptor pair analysis between macrophages and CD8$^+$ Tex in tumor tissue through Cellchat software. (F) Interaction strength between TREM2$^+$ macrophages and various types of CD8$^+$ T cells. (G)Interaction strength between CXCL and CD70 signaling pathways

among macrophage and $CD8^{+}$T cell subgroups. (H) CCL signaling pathway interactions between $CD8^{+}$ T cells and macrophages. (I) Dot-plot shows the results of the $TREM2^{+}$ Macrophage and $CD8^{+}$ T cell subgroups pair-receptor match calculation in the tumor tissue of our cohort, as determined by the CellphoneDB software (after p-values $<$ 0.05 filtering). (J) A strategy for mouse immune cell flow cytometry gating.

Figure S5

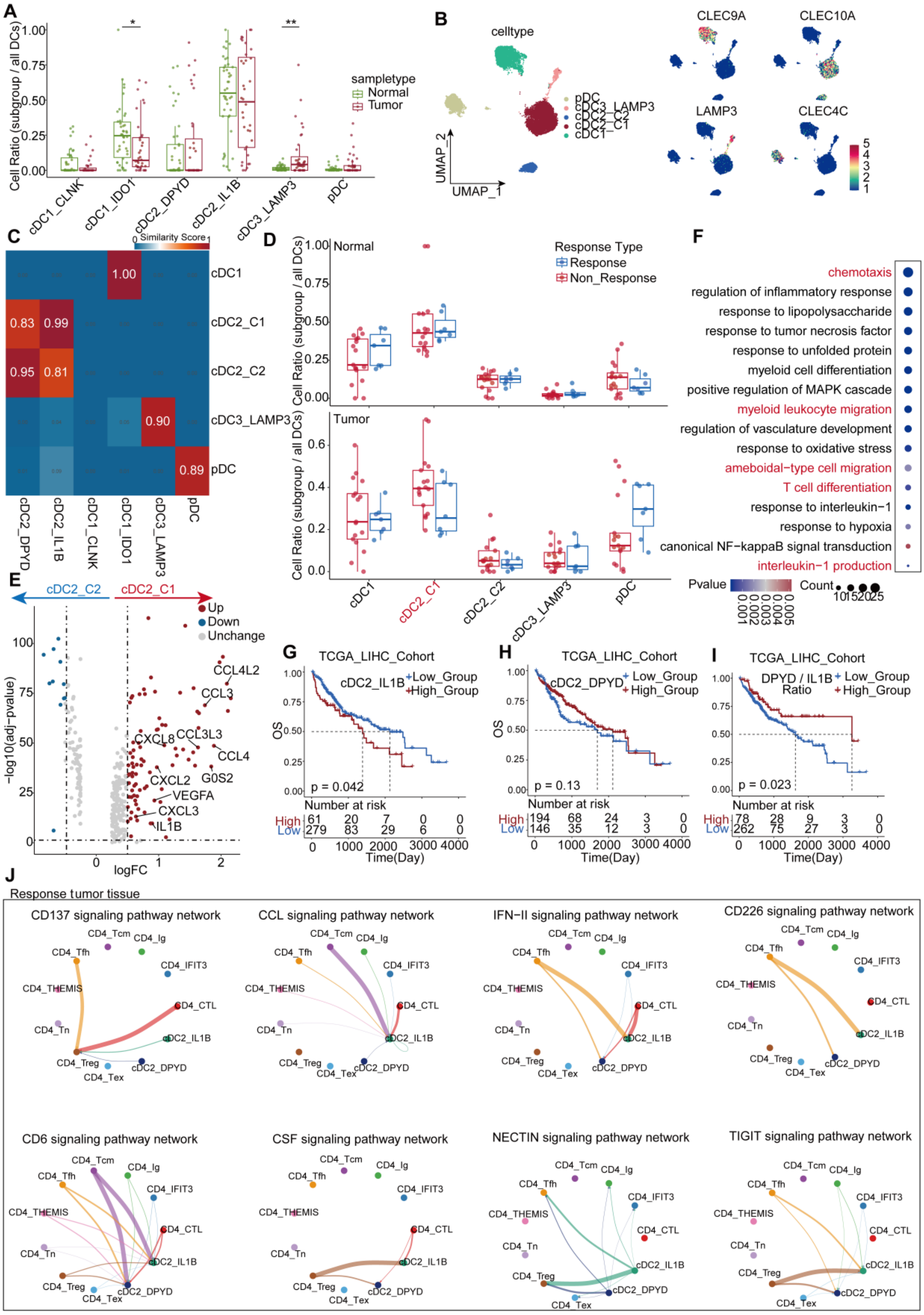

Figure S5. IL1B^{+} cDC2s are the main executor on cDC2s.

(A) Proportion statistics of DCs subgroups for individual patients across tumor and normal tissues. (Wilcoxon test. *P < 0.05, **P < 0.01, ***P < 0.001, ****P < 0.0001). (B) UMAP plot showing DCs sub-populations in the validation cohort. (C) The similarity score between dendritic cells in the validation cohort and discovery cohort. (D) The proportion of DCs classified by treatment response type in individual patients in normal tissue and tumor tissue.

(E) Volcano plot depicting differential genes between two cDC2 subgroups in the validation cohort. (F) Partial GO enrichment results for genes upregulated in IL1B^{+} cDC2 relative to DPYD^{+} cDC2. (G) Kaplan-Meier survival analysis of the IL1B^{+} cDC2 signature in the TCGA-LIHC cohort. (H) Kaplan-Meier survival analysis of the DPYD^{+}cDC2 signature in the TCGA-LIHC cohort. (I) Kaplan-Meier survival analysis of the DPYD^{+}cDC2 and IL1B^{+}cDC2 ratio in the TCGA-LIHC cohort. (J) Partial signaling pathway interactions between CD4^{+}T cells and cDC2 subgroups in tumor tissues of responsive patients.

Figure S6

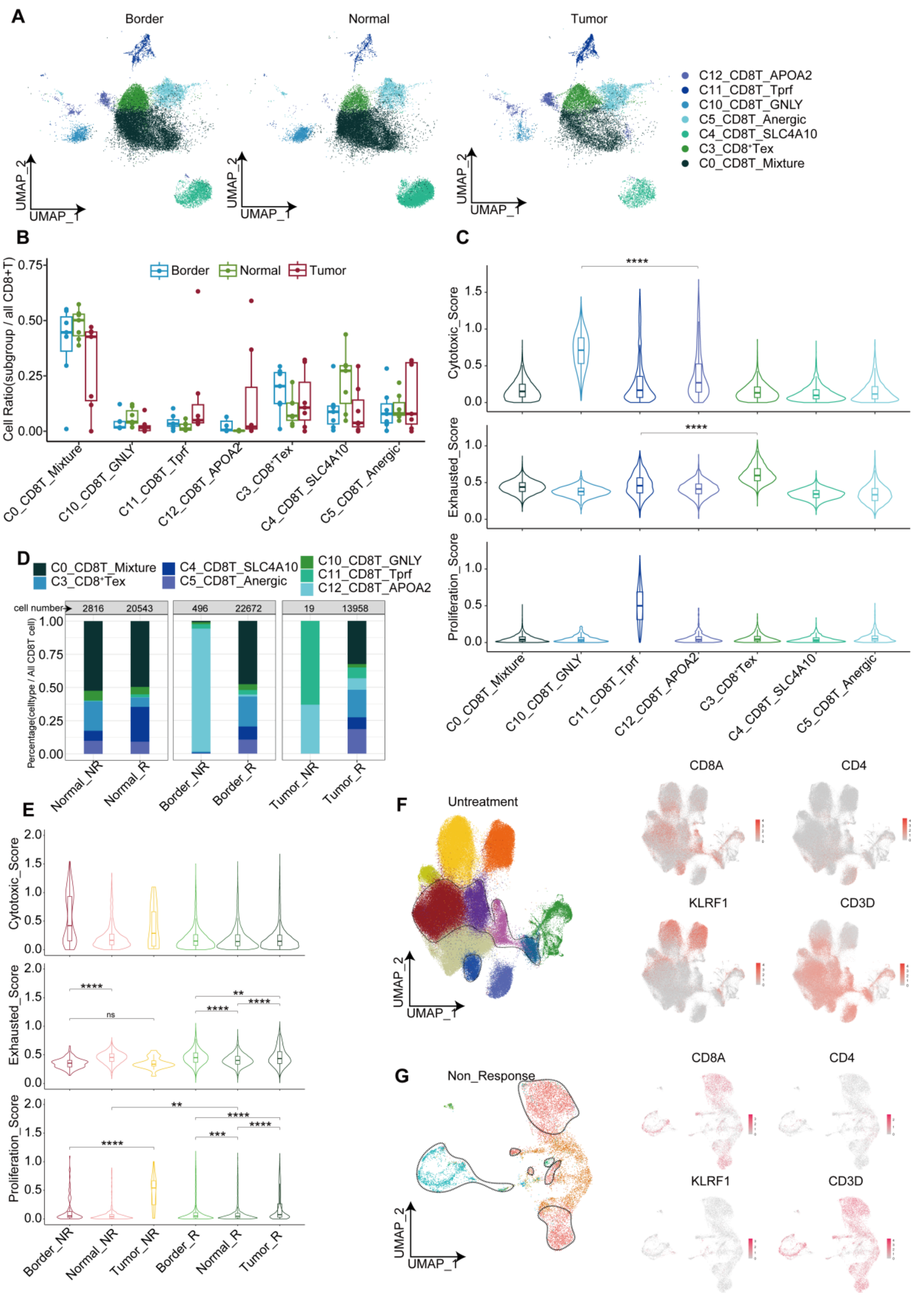

Figure S6. Transcriptional changes of CD8⁺ T cells after anti-PD-1 treatment.
UMAP plot showing the distribution of CD8⁺ T cells in the tumor, normal, and border area in this study cohort. (B) Proportion statistics of CD8⁺ T cell subgroups in the tumor, normal, and border areas for individual patients, showing their proportion among all CD8⁺ T cells. (C) Box and violin plots illustrating cytotoxicity, exhaustion, and proliferation scores for each CD8 ⁺T cell subgroup. (Wilcoxon test. *P ＜ 0.05, **P ＜ 0.01, ***P ＜ 0.001, ****P ＜ 0.0001). (D)

Proportion of $CD8^{+}$T cells across different tissue types and treatment conditions. (E) Box and violin plots showing cytotoxicity, exhaustion, and proliferation scores for $CD8^{+}$ T cells across different tissue types and treatment conditions. (Wilcoxon test. *P < 0.05, **P < 0.01, ***P < 0.001, ****P < 0.0001). (F-G) UMAP plot displaying expression levels of CD8A, CD4, KLRF1, and CD3D in NK/T cells in untreated patients.

Figure S7

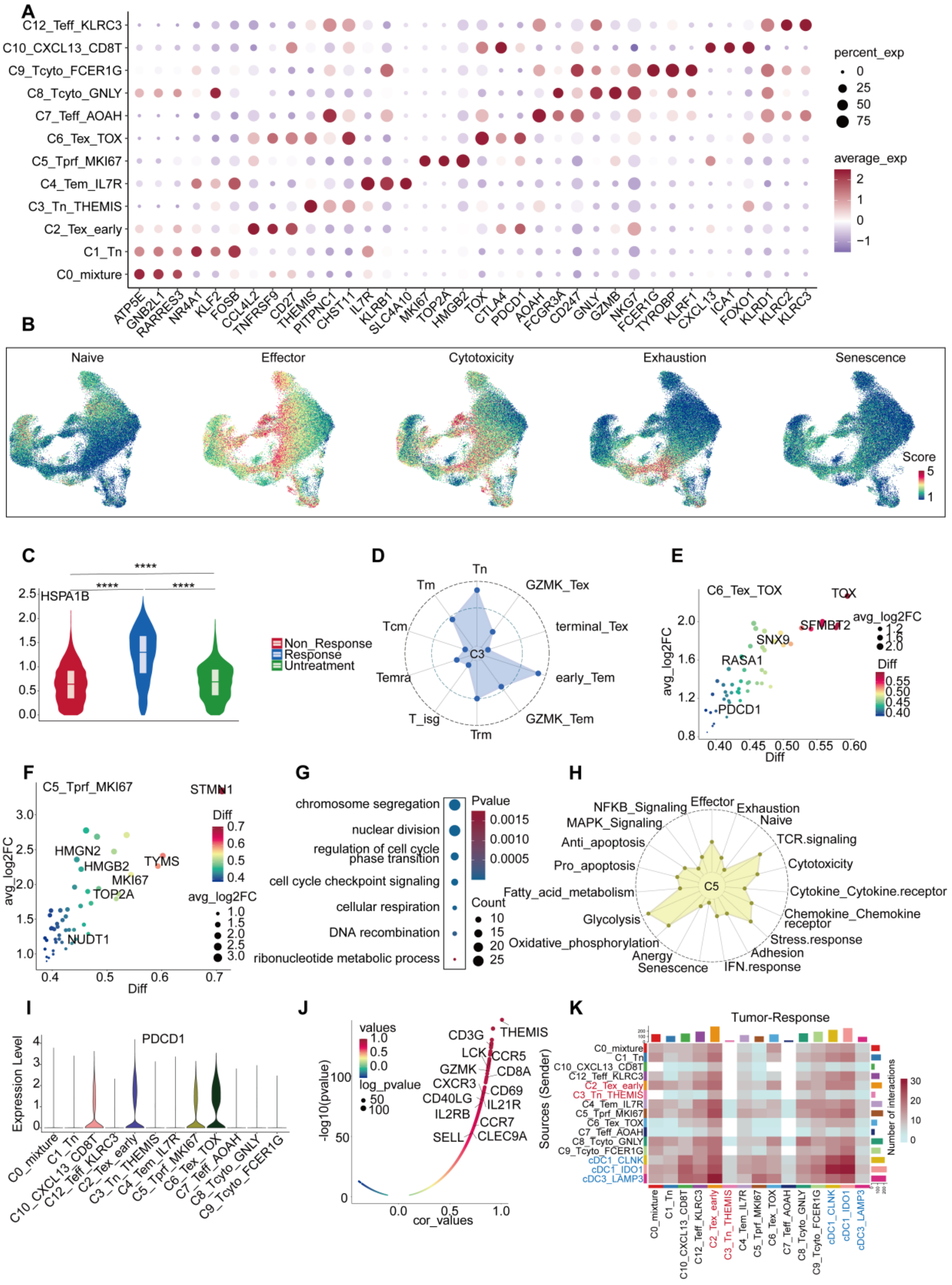

Figure S7. Transcriptional changes of CD8$^+$ T cells after anti-PD-1 treatment.

(A) Bubble plot showing markers corresponding to all CD8$^+$ T cell subgroups in the discovery cohort. (B) UMAP plot displaying CD8$^+$ T cell-related features. (C) Expression levels of HSPA1B in CD8$^+$ T cells with different treatment conditions. (Wilcoxon test. *P < 0.05, **P < 0.01, ***P < 0.001, ****P < 0.0001). (D) Radar plot illustrating cell type inclination scores for the C3_Tn_THEMIS subgroup. (E) Bubble plot displaying Top30 marker genes for the C6_Tex_TOX subgroup. (F)Bubble plot displaying Top30 marker genes for the C5_Tprf_MKI67 subgroup. (G) Partial GO enrichment results for marker genes of the

C5_Tprf_MKI67 subgroup. (H) Radar plot illustrating metabolism, signaling pathways, and functions of the C5_Tprf_MKI67 subgroup. (I) Expression levels of PDCD1 (PD-1) across different groups of $CD8^+$ T cells. (J) Dot plot showing genes highly correlated with THEMIS in the TCGA-LIHC cohort. (K) Heatmap showing the cell communication strength between $CD8^+$ T cells and certain dendritic cells in tumor tissues of responsive patients.

Figure S8

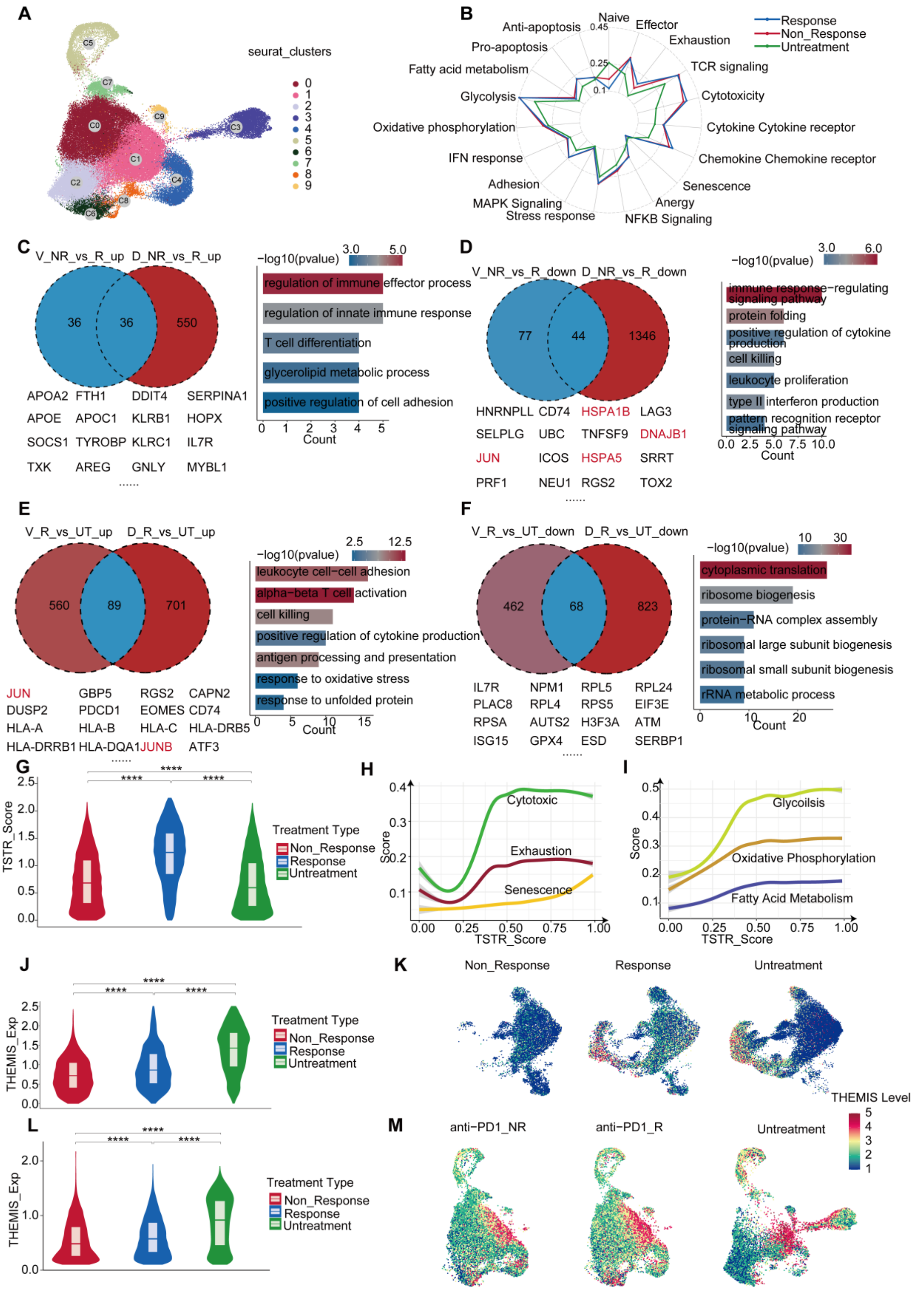

Figure S8. Transcriptional changes of CD8^{+} T cells after anti-PD-1 treatment.

UMAP plot showing the distribution of CD8^{+} T cells in the discovery cohort. (B) Radar plot illustrating metabolism, signaling pathways, and functions of CD8^{+} T cell in tumor tissue. (C-F) Venn diagram and bar plot showing the differentially expressed genes (in tumor tissue CD8^{+} T cells under different treatment conditions, log2FC > 0.5, p-values < 0.05) in the intersection of the discovery cohort. (D) and validation cohort (V) as well as the GO enrichment results of

these differentially expressed genes (NR: non-response, R: response). (G) TSTR signature level of $CD8^+$ T cell in different treatment conditions. (H-I) changes in cytotoxic, exhaustion, senescence, glucose metabolism, oxidative phosphorylation, and fatty acid metabolism score about TSTR scores. (J-K) THEMIS expression of $CD8^+$ T cell in different treatment conditions in the discovery cohort. (L-M) THEMIS expression of $CD8^+$ T cell in different treatment conditions in the validation cohort.

Figure S9

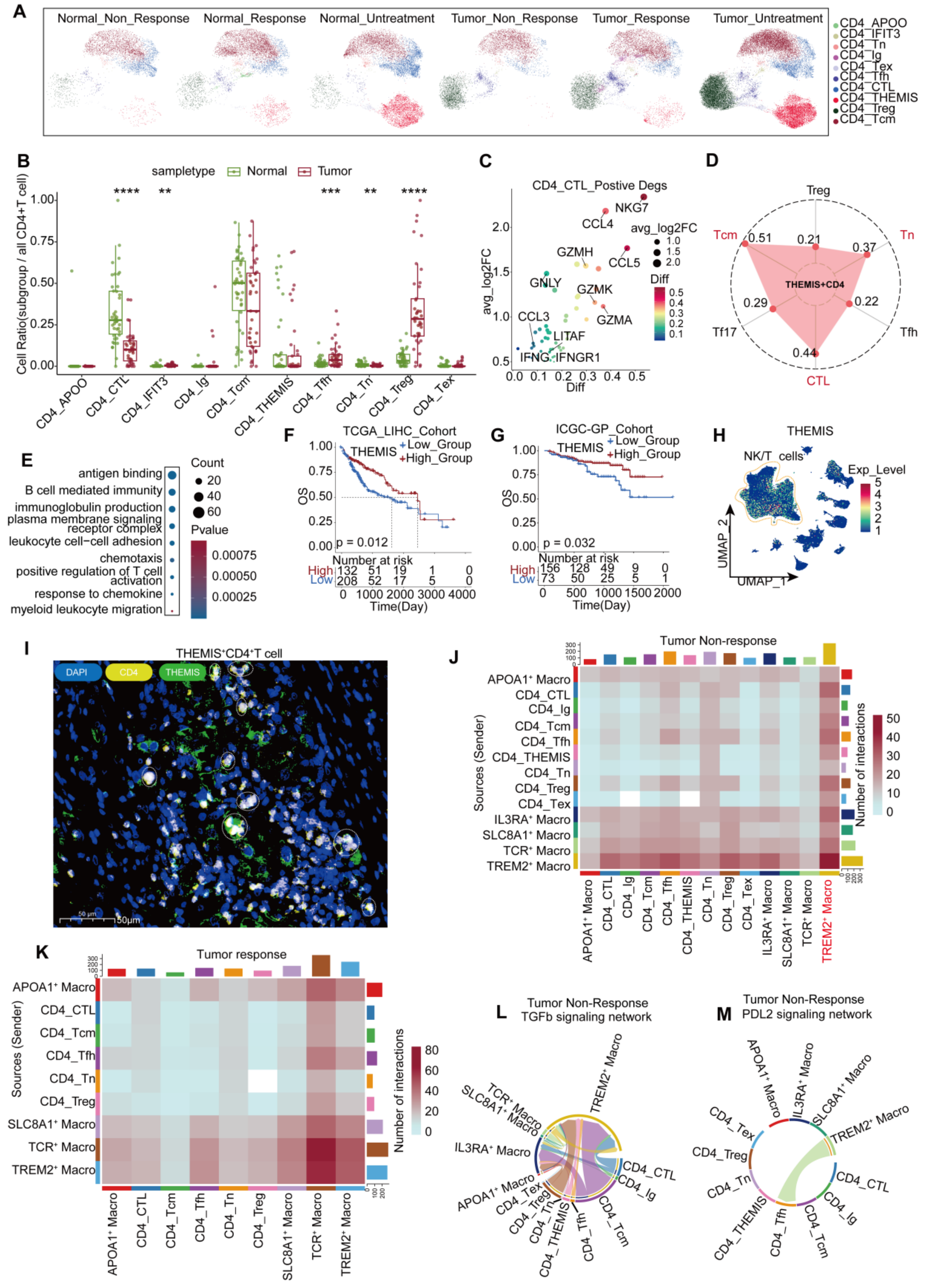

Figure S9. Transcriptional changes of $CD4^+$ T cells after anti-PD-1 treatment

(A) UMAP plot showing the distribution of $CD4^+$ T cells across tissue and treatment types after integration in the discovery cohort. (B) Proportion statistics of $CD4^+$ T cell subgroups in tumor and normal tissues for individual patients. (Wilcoxon test. *P < 0.05, **P < 0.01, ***P < 0.001, ****P < 0.0001). (C) Top 30 marker genes of CD4_CTL. (D) Radar plot displaying cell type inclination scores for $THEMIS^+$ CD4T. (E) Bubble plot showing partial GO enrichment results

for marker gene of the THEMIS$^+$ CD4T subgroup. (F) Kaplan-Meier survival analysis of THEMIS in the TCGA-LIHC cohort. (G) Kaplan-Meier survival analysis of THEMIS in the ICGC-JP cohort. (H) The expression levels of THEMIS in all cells indicate its primary expression in T cells. (I) Multicolor immunofluorescence validation of anti-PD-1 treatment efficacy, response, and non-response, as well as untreated patient tumor tissue THEMIS$^+$CD4$^+$ T cell density and statistical graphs. (J) Heatmap displaying cell communication strength between macrophages and CD4$^+$ T cells in tumor tissues of non-responsive patients. (K) Heatmap showing cell communication strength between macrophages and CD4$^+$ T cells in tumor tissues of responsive patients. (L) Communication of the TGF-β signaling pathway between macrophage subgroups and CD4$^+$ T cell subgroup in tumor tissues of non-responsive patients. (M) Communication of the PDL2 signaling pathway between macrophage and CD4$^+$ T cell subgroups in tumor tissues of non-responsive patients.

Figure S10

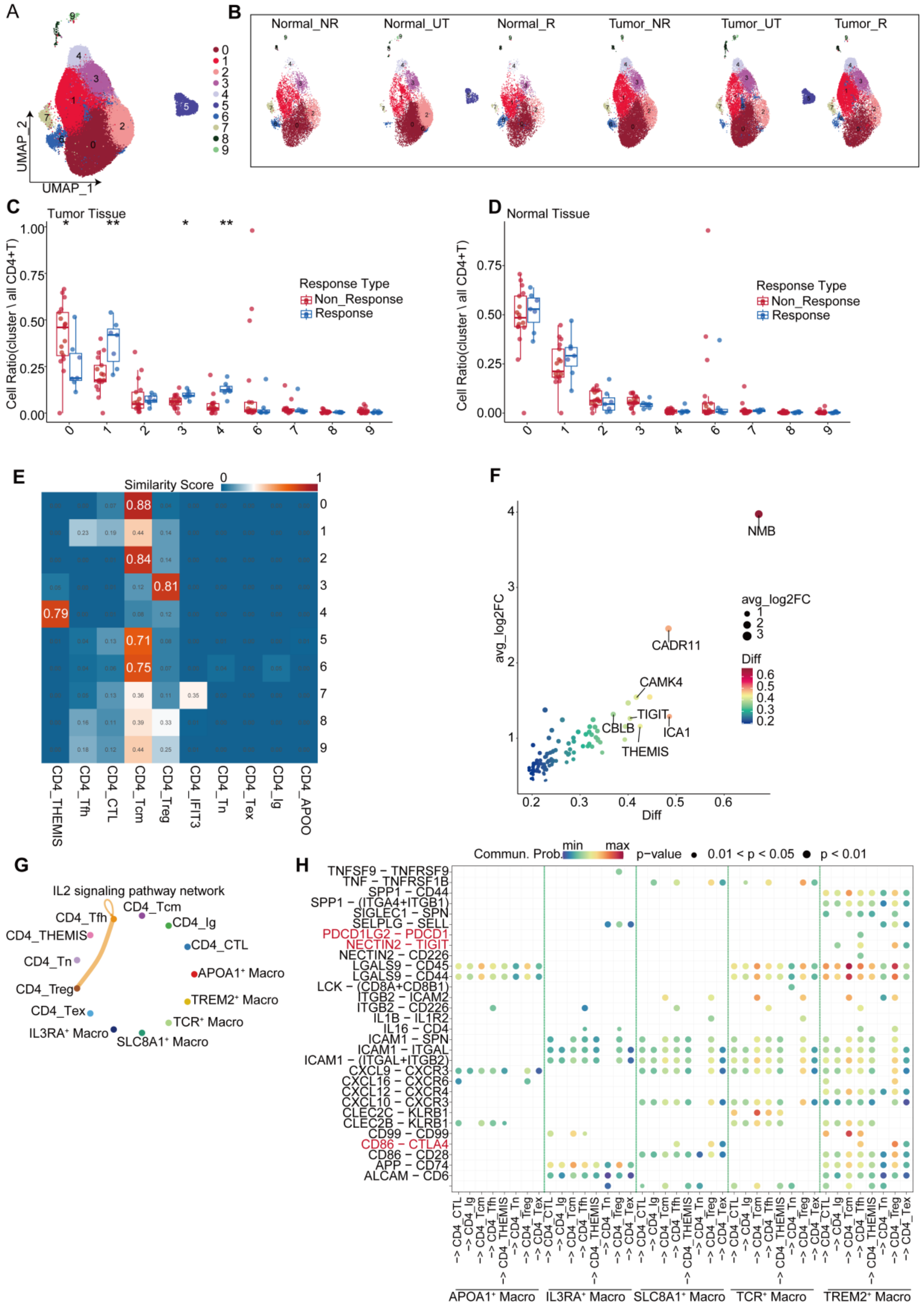

Figure S10. Transcriptional changes of CD4⁺ T cells after anti-PD-1 treatment

(A-B) UMAP plot showing the distribution of CD4⁺ T cells across tissue and treatment types in the validation cohort. (C-D) Proportion statistics of CD4⁺ T cell subgroups in tumor and normal tissues for individual patients in the validation cohort. (Wilcoxon test. *P < 0.05, **P < 0.01, ***P < 0.001, ****P < 0.0001). (E) The similarity score between the CD4⁺ T cell subgroup in the validation and discovery cohort. (F) marker genes of the C4-CD4⁺ T cluster in the

validation cohort. (G) Communication of the IL2 signaling pathway among $CD4^+$ T cell subgroups. (H) Bubble plot showing cell communication between various types of macrophages and $CD4^+$ T cells in the tumor microenvironment of non-responsive patients.
